# Time-resolved transcriptomic and proteomic profiling of *Heyndrickxia coagulans* during NaOH-buffered L-lactic acid production

**DOI:** 10.3389/fmicb.2023.1296692

**Published:** 2023-11-29

**Authors:** Xing Huang, Wenzhe Tian, Xiuwen Wang, Jiayang Qin

**Affiliations:** College of Pharmacy, Binzhou Medical University, Yantai, China

**Keywords:** *Heyndrickxia coagulans*, L-lactic acid, sodium hydroxide, time-resolved, transcriptomic and proteomic profiling

## Abstract

The L-lactic acid (L-LA) fermentation process, based on sodium hydroxide neutralization, demonstrates environmental friendliness during product extraction. However, lactate fermentation is hindered by the pronounced stress effect of sodium lactate on the strain compared with calcium lactate. In this study, we performed time-resolved transcriptomic and proteomic analyses of *Heyndrickxia coagulans* DSM1 during NaOH-buffered L-LA production. The expression levels of the glycolytic genes demonstrated an initial increase followed by a subsequent decrease, whereas the tricarboxylic acid cycle genes exhibited an initial decrease followed by a subsequent increase throughout the fermentation process. Moreover, we identified clusters of genes consisting of transcription factors and ATP-binding cassette (ABC) transporters that demonstrate a progressive elevation of expression levels throughout the fermentation process, with significant upregulation observed at later stages. This investigation yields valuable insights into the response mechanisms of *H. coagulans* during NaOH-buffered L-LA fermentation and presents potential targets for metabolic engineering.

## 1 Introduction

Lactic acid (LA), also known as dihydroxypropionic acid with a molecular formula of C_3_H_6_O_3_, is one of the primary organic acids globally (Juturu and Wu, 2016; Huang et al., 2021; Rawoof et al., 2021). Its versatile applications encompass diverse industries, including food, chemical, and pharmaceutical sectors. Of particular significance is its pivotal role in the synthesis of biodegradable materials such as polylactic acid (PLA) (Singhvi et al., 2019; Huang et al., 2021; Taib et al., 2023). PLA possesses exceptional mechanical properties along with biocompatibility and biodegradability, making it highly regarded within the industry as a promising novel packaging material. It is anticipated to gradually replace non-degradable materials derived from the petrochemical industry such as polyethylene, polypropylene, and polystyrene. This scenario will mitigate white pollution and alleviate the energy crisis (Upadhyaya et al., 2014; Klotz et al., 2016; Singhvi et al., 2019).

Currently, microbial fermentation is the predominant method utilized in the industrial production of LA (Huang et al., 2021; Rawoof et al., 2021). However, this process is characterized by product inhibition due to LA’s weak organic acid nature that lacks charge under low pH conditions (Singhvi et al., 2018a; Zhang et al., 2023). As a result, LA can easily permeate cell membranes through protonation and dissociation, causing toxic effects on cells and inducing significant stress (Singhvi et al., 2018a; Rawoof et al., 2021). Therefore, the addition of multiple buffers is commonly employed to maintain a neutral meta-acid pH level in the fermentation broth during industrial LA production. On the basis of the type of buffer used and resulting lactate in the fermentation broth, three distinct methods for LA production can be categorized: calcium lactate process (using calcium carbonate or calcium hydroxide as a buffer), sodium lactate process (utilizing sodium hydroxide or sodium carbonate as a buffer), and ammonium lactate process (employing ammonia as a buffer). The yields of LA fermentation and subsequent purification processes are significantly affected by various buffers employed.

The calcium lactate process is currently regarded as the most efficient and well-established method for LA production. However, a significant drawback of this technique is the necessity of adding sulfuric acid to facilitate LA purification, which leads to the generation of substantial industrial waste in the form of calcium sulfate (Juturu and Wu, 2016; Singhvi et al., 2018b). By contrast, the sodium lactate process has environmentally friendly characteristics. For instance, by implementing electrodialysis in sodium lactate solutions, LA purification can be achieved while simultaneously producing recyclable sodium hydroxide as a buffer. Consequently, this process eliminates any generation of industrial waste. Notably, membrane-based LA purification methods are not suitable for the calcium lactate process due to its tendency to form calcium hydroxide precipitates (Datta and Henry, 2006; Rawoof et al., 2021). The main challenge with the sodium lactate process lies in its low LA fermentation yield. Current research indicates that L-LA titer produced through the sodium lactate process generally remains around 100 g/L; this value is significantly lower than that achieved by the calcium lactate process, which is close to 200 g/L (Qin et al., 2010; Rawoof et al., 2021). Therefore, the response mechanism of LA-producing strains in relation to the sodium lactate process should be clarified.

In recent years, significant attention has been devoted to the production of L-LA by *Heyndrickxia coagulans*, formerly known as *Bacillus coagulans* or *Weizmannia coagulans* (Juturu and Wu, 2018; Zheng et al., 2018; Alves et al., 2023; Fu et al., 2023; Yang et al., 2023). Compared with traditional LA-producing strains such as *Lactobacillus* and *Rhizobium oryzae*, *H. coagulans* demonstrates superior characteristics including high optical purity of product, broad substrate spectrum, and non-sterilization fermentation (Fu et al., 2023; Yang et al., 2023). Currently, research on this strain primarily focuses on strain selection, optimization of fermentation conditions, and expansion of production raw materials. However, the response mechanism of *H. coagulans* in the sodium lactate process remains poorly understood. Therefore, this study aimed to elucidate the time-resolved response mechanism of *H. coagulans* DSM1 during NaOH-buffered L-LA fermentation via transcriptomic and proteomic approaches.

## 2 Materials and methods

### 2.1 Strain cultivation

*Heyndrickxia coagulans* DSM1, stored at −80°C, was inoculated onto LB solid medium (composed of 1% tryptone, 0.5% yeast extract, 0.5% NaCl, and 1.7% agar, pH range 6.7–7.0), and incubated at 50°C for 24 h to obtain individual colonies that were subsequently transferred to GY medium (containing 4% glucose, 1% yeast extract, and 0.1% CaCl_2_·2H_2_O, pH range of 6.7–7.0). The culture was further grown at a temperature of 50°C and agitation speed of 150 rpm for another day before being used as the inoculum in a fresh batch of GY medium. After an additional growth period of 8 h under the same conditions, the inoculum was introduced into a sterilized fermentation medium consisting of glucose (12%), yeast extract (1.5%), and CaCl_2_·2H_2_O (0.1 %) in a fermenter with controlled temperature at 50°C and stirring speed of 200 rpm but no ventilation. The pH was maintained at pH 6.0 with 10 M NaOH as buffer during the fermentation process. Measurements of strain growth, substrate consumption, and L-LA production were performed every 5 h. Samples collected at 5 h intervals from 5 h to 30 h were used for transcriptomic and proteomic analyses.

### 2.2 Transcriptomic studies

#### 2.2.1 Transcriptomic analysis

Transcriptomic analysis was conducted by using the Majorbio Cloud Platform (Ren et al., 2022). Gene expression quantification is determined by calculating the counts per kilobase length of transcripts per million (TPM) reads mapped. Significant differences in genes were identified based on predefined criteria, namely, |log_2_(FoldChange)| > 1 and *p*-value <0.05. Subsequently, differentially expressed genes (DEGs) underwent Gene Ontology (GO) and Kyoto Encyclopedia of Genes and Genomes (KEGG) enrichment analysis to elucidate dissimilarities in gene functions and metabolic pathways across various time points of the samples. To assess the enrichment of DEGs compared with all identified genes within each GO and KEGG category, we utilized a two-tailed Fisher’s exact test with standard false discovery rate control methods employed for correction. A corrected *p*-value <0.05 was considered statistically significant for both GO categories and KEGG pathways.

#### 2.2.2 RNA sequencing (RNA-seq)

The total RNA from *H. coagulans* DSM1 at different time points was extracted using TRIzol reagent from Invitrogen, followed by assessment of RNA integrity and the absence or presence of contamination through agarose gel electrophoresis. The purity of RNA was evaluated using a NanoDrop 8,000 Spectrophotometer (Thermo Scientific). Subsequently, mRNA isolation and purification were performed on the total RNA using the Ribo-Zero Magnetic Kit (Gram-positive bacteria), followed by the synthesis, purification, and construction of double-stranded cDNA. Finally, an Illumina sequencing platform (Illumina HiSeq 2000) was employed for RNA-Seq, which involved data processing, analysis, and standardization for further investigation. The sequencing data of *H. coagulans* DSM1 have been deposited in the NCBI Sequence Read Archive and can be accessed using the accession numbers SRR25442713 (5 h), SRR25442712 (10 h), SRR25442711 (15 h), SRR25442710 (20 h), SRR25442709 (25 h), and SRR25442708 (30 h).

#### 2.2.3 Quantitative real-time PCR (qRT-PCR) verification

Four genes belonging to four different operons were chosen to confirm RNA-seq data by qRT-PCR. The PCR conditions were implemented as follows: 95°C for 10 s and 60°C for 30 s in 40 repeated cycles. The 16S rRNA gene was selected as the reference gene (Qin et al., 2015). The genes and primers used for qRT-PCR are shown in Supplementary Table S1. The relative gene expression data were analyzed via the 2^-ΔΔCt^ method. All qRT-PCR runs were conducted with three biological and three technical replicates.

### 2.3 Quantitative proteomic studies

#### 2.3.1 Protein extraction

Each sample was transferred to a 5 mL centrifuge tube and subjected to three rounds of sonication on ice using a high-intensity ultrasonic processor in lysis buffer containing 8 M urea, 2 mM ethylene diamine tetraacetic acid (EDTA), 10 mM dithiothreitol (DTT), and 1% Protease Inhibitor Cocktail III (Merk). The resulting debris was eliminated through centrifugation at 20,000 g at 4°C for 10 min. Subsequently, protein precipitation was achieved by adding cold 15% trichloroacetic acid followed by incubation at −20°C for 2 h. After another round of centrifugation at 4°C for 10 min, the supernatant was discarded. The remaining precipitate underwent three washes with cold acetone. Protein reconstitution was performed using a buffer solution consisting of 8 M urea and 100 mM tetraethylammonium bromide (TEAB), with the pH adjusted to pH 8.0. Finally, the protein concentration was determined using the manufacturer’s guidelines provided with the 2-D Quant kit.

#### 2.3.2 Trypsin digestion and TMT labeling

The protein solution was subjected to a reduction process using 10 mM DTT for 1 h at 37°C, followed by alkylation with 20 mM iodoacetamide (IAA) for 45 min at room temperature in darkness. To facilitate trypsin digestion, the protein sample was diluted by adding 100 mM TEAB to achieve a urea concentration below 2 M. Subsequently, trypsin was added at a trypsin-to-protein mass ratio of 1:50 for the initial overnight digestion and a ratio of 1:100 for the subsequent 4 h digestion period. Each sample contained approximately 100 μg worth of proteins that underwent trypsin digestion. After digestion, peptides were desalted using a Strata X C18 solid-phase extraction column (Phenomenex) and then vacuum dried. The desalted peptides were reconstituted in a solution of 0.5 M TEAB and processed following the manufacturer’s protocol for the Thermo America’s TMT kit (6-plex). In brief, one unit of TMT reagent (defined as the quantity required to label 100 μg of protein) was thawed and reconstituted in acetonitrile (ACN) (24 μL). The peptide mixtures were incubated at room temperature for 2 h, pooled together, desalted, and dried using vacuum centrifugation.

#### 2.3.3 HPLC fractionation

The sample was fractionated into 80 fractions using high pH reverse-phase HPLC with an Agilent 300Extend C18 column (5 μm particles, 4.6 mm ID, and 250 mm length) by separating peptides with a gradient of 2 to 60% acetonitrile in 10 mM ammonium bicarbonate at pH 10 over a duration of 80 min. The resulting fractions were then consolidated into 18 and dried through vacuum centrifugation.

#### 2.3.4 LC–MS/MS analysis

The peptides were dissolved in a solution containing 0.1% formic acid (FA) and loaded onto a reversed-phase pre-column (Acclaim PepMap 100, Thermo Scientific). Peptide separation was performed using a reversed-phase analytical column (Acclaim PepMap RSLC, Thermo Scientific) with a gradient elution of solvent B (0.1% FA in 98% acetonitrile), starting at 6% and increasing to 25% over 26 min, followed by an increase from 25 to 40% over the next 8 min, and finally reaching 80% in 3 min. The flow rate was maintained at a constant of 450 nL/min using an EASY-nLC 1,000 UPLC system. Subsequently, the resulting peptides were analyzed using Q Exactive^™^ plus hybrid quadrupole-Orbitrap mass spectrometer (ThermoFisher Scientific).

#### 2.3.5 Database search

The peptides were subjected to NSI source and subsequent tandem mass spectrometry (MS/MS) analysis using a Q Exactive^™^ plus instrument (Thermo) coupled online to the UPLC system. Intact peptides were identified in the Orbitrap at a resolution of 70,000. Peptides meeting specific criteria were selected for MS/MS analysis using an NCE setting of 30, and ion fragments were detected in the Orbitrap at a resolution of 17,500. A data-dependent method was employed, consisting of one MS scan followed by 20 MS/MS scans targeting the top 20 precursor ions with an ion count threshold of 10,000 in the MS survey scan. To prevent excessive filling of the ion trap, we utilized automatic gain control (AGC), resulting in the accumulation of up to 5E4 ions for generating high-quality MS/MS spectra. The m/z range during MS scans was set from 350 to 1800 with a fixed first mass value of 100 m/z while employing an electrospray voltage of 2.0 kV.

#### 2.3.6 Proteomic analysis

Proteomic analysis was performed using the Majorbio Cloud Platform (Ren et al., 2022) and differentially expressed proteins (DEPs) were identified. The DEPs were subjected to GO and KEGG enrichment analyses, following the same criteria as described in section 1.2.3.

### 2.4 Time-resolved transcriptomic and proteomic analyses

A time-series analysis utilizing STEM software was performed on samples collected at multiple time points to investigate temporal changes in gene and protein expression (Ernst and Bar-Joseph, 2006). Gene and protein expression levels were normalized through log2 transformation. Specifically, we calculated the fold change (FC) in expression relative to the initial time point for all samples before taking its logarithm base 2 value. After normalizing these data accordingly, we designated an expression level of zero for the first sample; subsequent samples with positive values indicated up-regulation of genes/proteins while negative values suggested down-regulation. Through trend analysis techniques employed here afterwards, we successfully identified co-expressed genes sharing similar patterns of differential regulation over time.

## 3 Results and discussion

### 3.1 NaOH-buffered L-LA fermentation by *Heyndrickxia coagulans* DSM1

**Figure 1:**
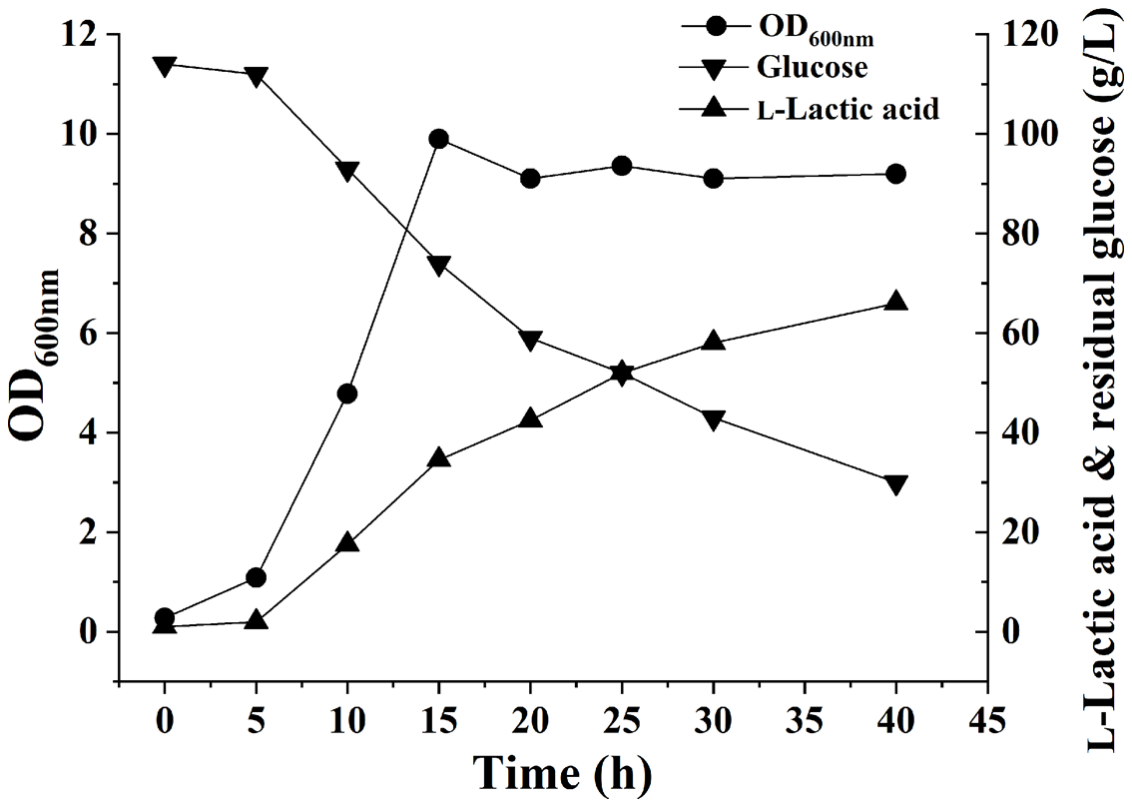
Fermentation process curve of *H. coagulans* DSM1.

The pH of the fermentation liquid was adjusted to 6.0 by adding NaOH as a buffer, and *H. coagulans* DSM1 was utilized for L-LA fermentation. Cell growth, residual glucose (RG), and L-LA concentrations were evaluated at 5-h intervals using the previously established methods (Tian et al., 2022). After 5 h, cell growth entered the exponential phase with an OD_600_ value reaching 9.9 by the 15th hour. Subsequently, the OD_600_ value stabilized at approximately 9. The L-LA titer exhibited a rapid increase within 5 h, reaching 42.5 g/L by 15 h. Thereafter, it showed a gradual increase and reached 58 g/L by the end of the experiment (Figure 1). The growth curve of strain and L-LA production revealed that noticeable product inhibition occurred after 15 h. To investigate *H. coagulans*’ stress response mechanism during L-LA production, we extracted RNA and total protein at six specific time points (5, 10, 15, 20, 25, and 30 h), followed by RNA-Seq and quantitative proteomic analysis.

### 3.2 Comparative transcriptomic analysis

**Figure 2:**
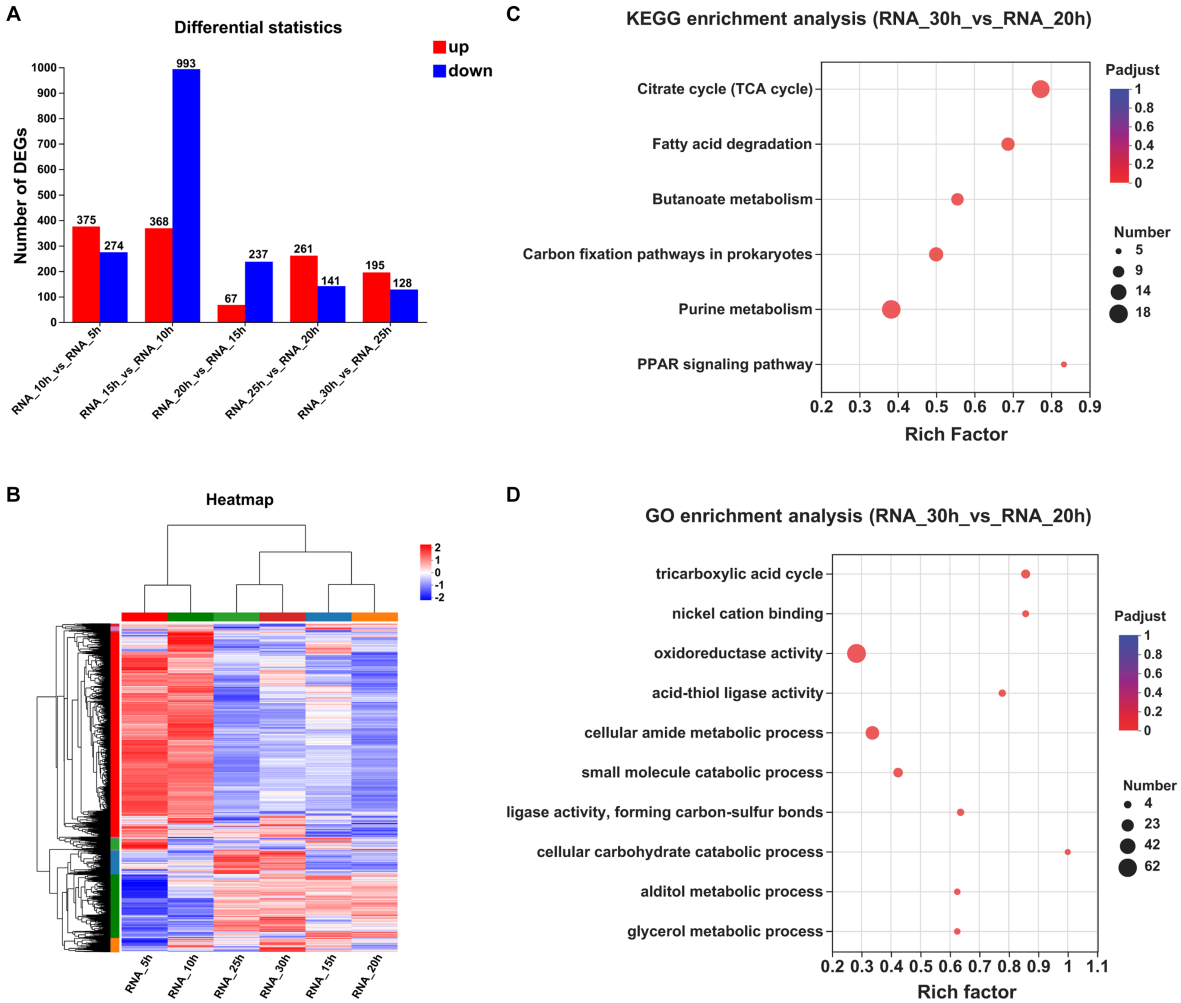
Comparative transcriptomic analysis of samples at different time points. **(A)** The numbers of DEGs observed at a 5 h interval. **(B)** Clustering heatmap of the genes at a 5 h interval. **(C)** KEGG enrichment analysis between the samples collected at 30 and 20 h (*P_adjust_* < 0.05). **(D)** GO enrichment analysis between the samples collected at 30 and 20 h (*P_adjust_* < 0.05).

The correctness of the transcriptome results was verified by qRT-PCR (Supplementary Table S2). The numbers of DEGs observed at a 5 h interval are presented in Figure 2A. Specifically, between the 10 and 5 h samples, a total of 649 DEGs were identified, with 375 upregulated and 274 downregulated. Similarly, between the 15 and 10 h samples, a total of 1,361 DEGs were detected, with 368 upregulated and 993 downregulated. Furthermore, between the 20 and 15 h samples, a total of only 304 DEGs were observed, of which 67 were upregulated genes and 237 were downregulated genes. Additionally, between the 25 and 20 h samples, a total of 402 DEGs were identified, with 261 upregulated genes and 141 downregulated genes. Finally, between the 30 and 25 h samples, a total of 323 DEGs were found, with 195 upregulated genes and 128 downregulated genes. The clustering heatmap of the genes is depicted in Figure 2B.

To investigate temporal changes in gene expression levels upon entry of strain growth into a stationary phase, we performed KEGG and GO enrichment analyses on DEGs across multiple time points. Only one KEGG pathway exhibited significant enrichment between samples collected at 20 and 15 h, and 19 pathways showed significant enrichment between samples collected at 25 and 20 h, with an additional 4 pathways displaying significant enrichment between samples obtained at 30 and 25 h (Supplementary Table S3). By extending the time interval to 10 h, we observed significant enrichment of six KEGG pathways between the samples collected at 30 and 20 h: “citrate cycle,” “fatty acid degradation,” “butanoate metabolism,” “carbon fixation pathways in prokaryotes,” “purine metabolism,” and the “PPAR signaling pathway” (Figure 2C).

The GO enrichment analyses of the DEPs revealed a total of 84 significantly regulated GO terms between the samples collected at 20 and 15 h, 98 significantly regulated GO terms between the samples collected at 25 and 20 h, and 54 significantly regulated GO terms observed between the samples collected at 30 and 25 h. By extending the time interval to 10 h, we observed significant enrichment in a total of 66 GO terms between the samples collected at 30 and 20 h. Notably, among these enriched terms, “tricarboxylic acid (TCA) cycle,” “nickel cation binding,” “oxidoreductase activity,” “acid-thiol ligase activity,” and “cellular amide metabolic process” emerged as the top five significantly regulated GO terms (Figure 2D).

The top 20 genes exhibiting a significant increase in gene expression in the 30 h samples compared with the 20 h samples are presented in Supplementary Table S4. Among these, five transposase family proteins (BF29_RS04425, BF29_RS09895, BF29_RS12685, BF29_RS16355, and BF29_RS02245) showed significantly elevated levels of gene expression. Additionally, an aquaporin family protein (BF29_RS13625) demonstrated substantial upregulation. The protein sequence of BF29_RS13625 shared a similarity of 72% with glycerol uptake facilitator protein (P18156) found in *Bacillus subtilis* 168, suggesting its potential role in facilitating glycerol transport. Furthermore, upregulation was observed for BF29_RS14480, which shared a sequence identity of 67.1% with L-lactate permease (P71067) of *B. subtilis* 168 known as the primary permease responsible for L-lactate uptake (Chai et al., 2009). Several other transporters also exhibited significant increases including glycoside-pentoside-hexuronide (GPH): cation symporter (BF29_RS06670), MFS transporter (BF29_RS16105), and sugar ABC transporter permease (BF29_RS14615). Moreover, we noted a substantial increase observed in the expression level of the AraC family transcription factor (BF29_RS14625).

### 3.3 Comparative proteomic analysis

**Figure 3:**
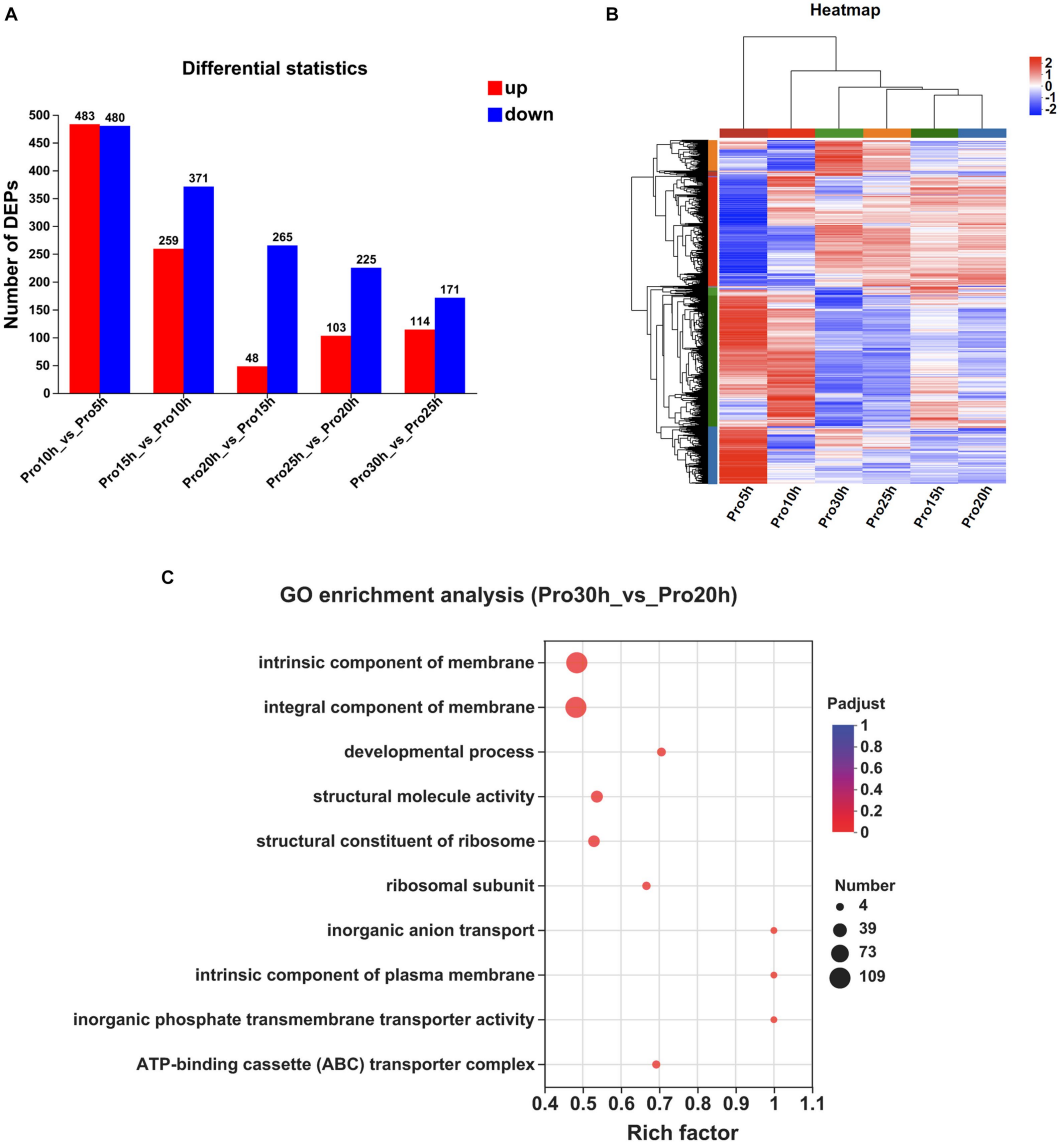
Comparative proteomic analysis of samples at different time points. **(A)** The number of DEPs observed at a 5 h interval. **(B)** Clustering heatmap of the genes at a 5 h interval. **(C)** GO enrichment analysis between the samples collected at 30 and 20 h (*P_adjust_* < 0.05).

The numbers of DEPs observed at a 5 h interval are presented in Figure 3A. A total of 963 DEPs were identified between the 10 and 5 h samples, with 483 upregulated and 480 downregulated. Similarly, 630 DEPs were identified between the 15 and 10 h samples, consisting of 259 upregulated and 371 downregulated proteins. Furthermore, the comparison between the 20 and 15 h samples revealed 313 DEPs, with 48 up-regulated and 265 down-regulated proteins. The 25 and 20 h samples exhibited 328 DEPs, including 103 upregulated and 225 downregulated proteins. Lastly, the 30 and 25 h samples showed 285 DEPs, with 114 upregulated and 171 downregulated proteins. The clustering heatmap of the proteins is presented in Figure 3B.

The DEPs from multiple time points were subjected to KEGG and GO enrichment analyses to investigate protein expression fluctuations during the stable growth phase of strains. No significant enrichment in any KEGG pathway was observed between samples collected at 20 and 15 h, as well as between samples collected at 25 and 20 h. Only one KEGG pathway, namely, “Ribosome,” exhibited significant enrichment between samples collected at 30 and 25 h. When extending the time interval to 10 h, no significant enrichment in any KEGG pathway was observed between samples collected at 30 and 20 h.

The GO enrichment analyses of the DEPs identified a total of 48 significantly regulated GO terms between the samples collected at 20 and 15 h, 41 significantly regulated GO terms between the samples collected at 25 and 20 h, and 46 significantly regulated GO terms observed between the samples collected at 30 and 25 h. By extending the time interval to 10 h, we observed significant enrichment in a total of 32 GO terms between the samples collected at 30 and 20 h. Notably, among these enriched terms, “intrinsic component of membrane,” “integral component of membrane,” “developmental process,” “structural molecule activity,” and “structural constituent of ribosome” emerged as the top five significantly regulated GO terms (Figure 3C).

The top 10 proteins exhibiting a significant increase in protein expression in the 30 h samples compared with the 20 h samples are displayed in Supplementary Table S5. Among these identified proteins, three transporters were observed, namely, BF29_RS01725, BF29_RS03835, and BF29_RS06895. BF29_RS01725 is classified as an MFS transporter and shares a protein sequence identity of 40% with the 2-nitroimidazole transporter (P76242) discovered in *Escherichia coli* K-12, suggesting its potential involvement in facilitating the transport of 2-nitroimidazole. Both BF29_RS03835 and BF29_RS06895 are classified as ABC transporters. The former exhibits a protein sequence identity of 57% with zinc uptake system ATP-binding protein ZurA (Q9XDA6) of *Listeria monocytogenes* EGD-e, whereas the latter shares a sequence identity of 75% with dipeptide transport system permease protein DppC (P26904) of *B. subtilis* 168. Notably, CvpA family protein (BF29_RS04995), which potentially plays a role in colicin synthesis (Skvirsky et al., 1996), was significantly upregulated.

### 3.4 Integration of transcriptomic and proteomic analyses

The correlation between transcriptomic and proteomic data was compared, revealing an overlap of 1,641 mappings between proteins and genes. A total of 771 distinct genes were identified in the transcriptome analysis, whereas 65 unique proteins were identified in the proteome analysis. The results of GO and KEGG enrichment analyses of the transcriptome and proteome were compared. The comparison between the 30 and 20 h samples revealed significant enrichment of four GO terms in both transcriptome and proteomics analyses, namely, “phosphate ion transport,” “structural molecule activity,” “structural constituent of ribosome,” and “inorganic phosphate transmembrane transporter activity” (Figure S1). However, no KEGG pathways between the 30 and 20 h samples were significantly enriched in both transcriptome and proteome analyses (*P_adjust_* < 0.05).

A set of 40 genes exhibited significant up-regulation in the 30 h samples compared with the 20 h samples as determined by both transcriptomic and proteomic analyses (Supplementary Table S6). Notably, BF29_RS13625 and BF29_RS16635 ranked among the top 20 in terms of gene expression level changes mentioned earlier. BF29_RS13625, annotated as a glycerol uptake facilitator protein, was also ranked among the top 20 alterations in protein expression. BF29_RS13625 is located adjacent to BF29_RS13630 and BF29_RS13635 in the genome, which, respectively, encode glycerol kinase GlpK and glycerol-3-phosphate dehydrogenase. These three genes play a crucial role in the processes of glycerol uptake, phosphorylation, and dehydrogenation, facilitating the conversion of glycerol into dihydroxyacetone phosphate – an intermediate compound involved in glycolysis (Doi, 2019). Although glycerol is not present in the fermentation medium, it serves as a key metabolite in various pathways and can be derived from multiple sources including glucose, proteins, pyruvate, triacylglycerols, and other glycerolipid metabolic pathways. Intracellular glycerol can be transported outside of the cell through specialized transport systems to maintain a balance between intracellular and extracellular levels. The uptake and synthesis of compatible solutes are microbial stress responses to high-osmolality environments (Brisson et al., 2001). Therefore, the changes in the expression of glycerol metabolism genes may serve as a responsive mechanism of *H. coagulans* DSM1 to environmental fluctuations during the late stage of fermentation.

Additionally, 19 genes showed significant down-regulation in the 30 h samples compared with the 20 h samples based on both transcriptomic and proteomic analyses (Supplementary Table S7). Of these genes, BF29_RS10675 displayed the highest fold downregulation at both gene and protein levels. Interestingly, BF29_RS10675 belonged to ABC transporter ATP-binding protein family and shares a protein sequence identity of 64.1% with *Pullulanibacillus camelliae*’s glycerol-3-phosphate ABC transporter ATP-binding protein (A0A8J2VFZ8), suggesting its potential role in facilitating glycerol-3-phosphate transport.

### 3.5 Time-resolved transcriptomic and proteomic analyses

**Figure 4:**
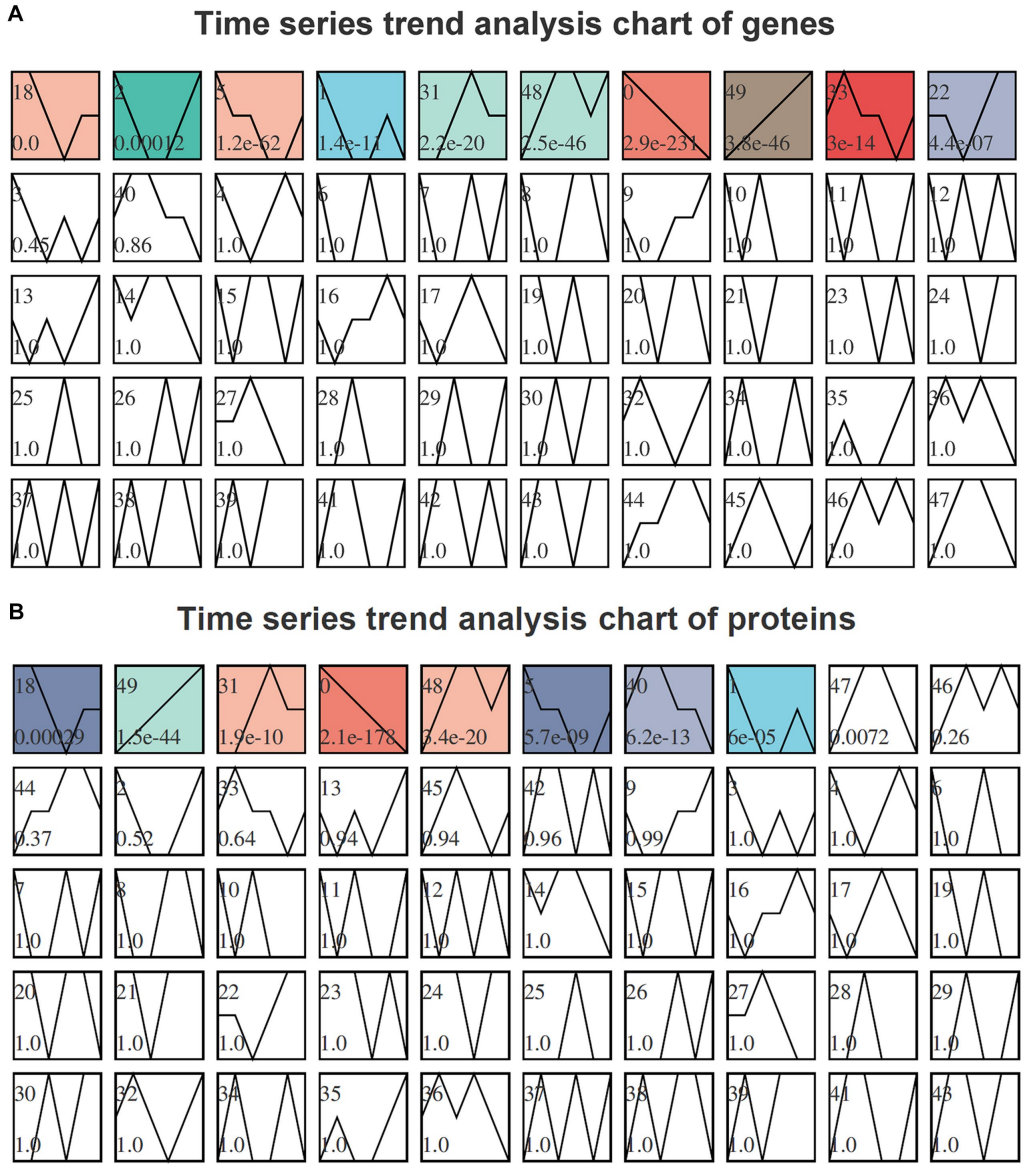
Time-series trend analysis of genes **(A)** and proteins **(B)** by STEM. The module number is indicated in the upper left corner of each module. The trend line represents the curve fitting of genes or proteins within each module. The *p*-value, located in the lower left corner of the module, signifies the level of enrichment significance. Modules colored as *p* < 0.05 indicate significantly enriched modules. Genes or proteins exhibiting similar trends and sharing identical module colors can be categorized together for further analysis.

To analyze the trends in gene expression during fermentation, we performed STEM time-series analysis. The results on DEGs revealed a significant enrichment in modules 18, 2, 5, 1, 31, 48, 0, 49, 33, and 22. Notably, module 49 exhibited a gradual increase in gene expression levels over time, as depicted in Figure 4A. Similarly, the STEM time-series analysis on DEPs demonstrated significant enrichment in modules 18, 49, 31, 0, 48, 5, 40, and 1. Among these modules, a progressive increase was observed in protein expression levels over time for module 49 as illustrated in Figure 4B.

**Figure 5:**
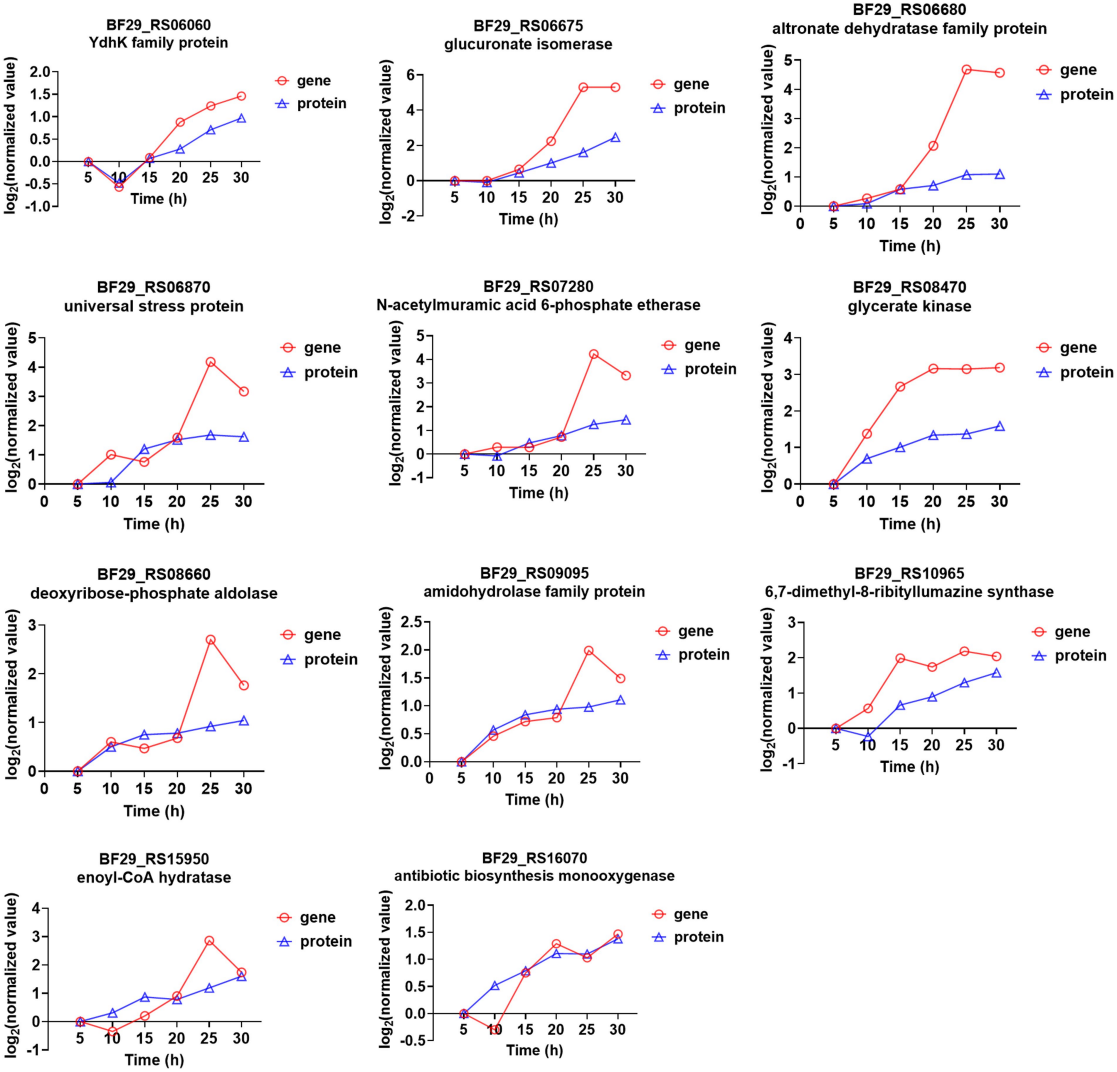
Genes that were identified in module 49 through both transcriptome and proteome time-series analyses and their corresponding expression trends.

In the transcriptome time-series analysis, module 49 comprised a total of 153 known genes, including 3 novel genes and 1 sRNA (Supplementary Table S8). Similarly, in the proteomic time-series analysis, module 49 encompassed a set of 70 known proteins (Supplementary Table S9). By intersecting the results from both transcriptome and proteome analyses, a total of 11 genes were identified to exhibit consistent expression patterns over time (Figure 5). Among these genes, BF29_RS06675 and BF29_RS06680 belonged to the same operon, which consisted of six genes (the other four being BF29_RS06670, BF29_RS06685, BF29_RS06690, and BF29_RS06695). Their expression patterns exhibited a significant upregulation after 15 h. These genes were involved in KEGG pathways related to “Pentose and glucuronate interconversions” and “Pentose phosphate pathway,” playing a crucial role in the degradation of D-glucuronate into pyruvate and glyceraldehyde 3-phosphate.

BF29_RS07280 is a member of an operon comprising four genes (BF29_RS07270, BF29_RS07275, and BF29_RS07285), which are predicted to be involved in peptidoglycan recycling. These genes were also found to exhibit significant upregulation after 15 h. Peptidoglycan recycling refers to the bacterial importation of cell wall degradation products for their incorporation into either peptidoglycan biosynthesis or basic metabolic pathways (Hadi et al., 2008). Specifically, BF29_RS07280 is predicted to encode N-acetylmuramic acid 6-phosphate etherase, responsible for catalyzing the hydrolysis of N-acetylmuramic acid 6-phosphate (MurNAc 6-phosphate) into N-acetylglucosamine-6-phosphate (GlcNAc 6-phosphate), an essential component of the cell wall.

### 3.6 Time-resolved expression analysis of key enzymes in the L-LA metabolic pathway

**Figure 6:**
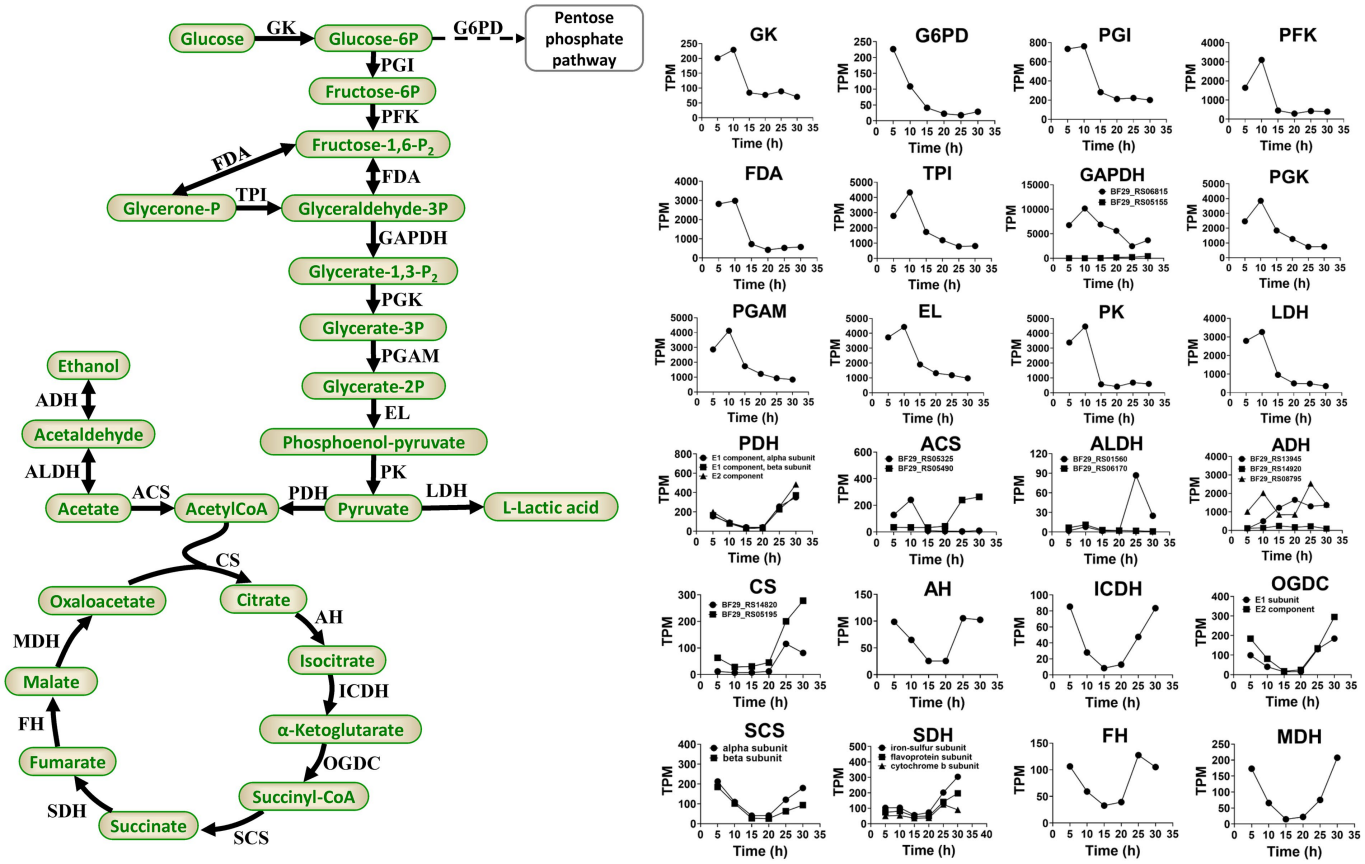
Primary pathways involved in L-LA metabolism of *H. coagulans* DSM1 and the temporal dynamics of gene expression levels for key enzymes. GK, glucokinase; G6PD, glucose-6-phosphate dehydrogenase; PGI, glucose-phosphate isomerase; PKF, phosphofructokinase; FDA, fructose 1,6-bisphosphate aldolase; TPI, triose phosphate isomerase; GAPDH, glyceraldehyde-3-phosphate dehydrogenase; PGK, phosphoglycerate kinase; PGAM, mitochondrial glycero-3-phosphoacyltransferase; EL, enolase; PK, pyruvate kinase; LDH, L-lactate dehydrogenase; PDH, pyruvate dehydrogenase; CS, citrate synthase; AH, aconitate hydratase; ICDH, isocitrate dehydrogenase; OGDC, 2-oxoglutarate dehydrogenase complex; SCS, succinyl-coa synthetase; SDH, succinate dehydrogenase; FH, fumarate hydratase; MDH, malate dehydrogenase; ACS, acetyl-coa synthetase; ALDH, aldehyde dehydrogenase; and ADH, alcohol dehydrogenase.

During the fermentation process of *H. coagulans*, glucose is phosphorylated to form glucose 6-phosphate. Subsequently, this compound undergoes isomerization to fructose 6-phosphate, followed by decomposition through phosphorylation and aldolase reactions, resulting in the production of glyceraldehyde 3-phosphate. The latter is then catalyzed by dehydrogenase and oxidized to 1,3-diphosphoglycerate in the presence of NAD^+^ and H_3_PO_4_. Phosphoenolpyruvate is subsequently generated through the catalytic actions of phosphoglycerate kinase, phosphoglycerate mutase, and enolase. A fraction of pyruvate derived from phosphoenolpyruvate is enzymatically converted into L-LA by L-lactate dehydrogenase, whereas the remaining portion enters the TCA cycle (Figure 6).

Sodium lactate induces higher levels of stress on *B. coagulans* compared with calcium lactate (Wang et al., 2014; Juturu and Wu, 2016). Additionally, sodium lactate stress downregulates the expression levels of glycolysis genes but upregulates the expression levels of TCA cycle genes in *B. coagulans* 2–6. Conversely, calcium lactate stress upregulates glycolysis genes but exerts minimal impact on the expression levels of TCA cycle genes (Qin et al., 2015). In this study, Figure 6 depicts the temporal dynamics of gene expression levels for crucial enzymes involved in glycolysis and TCA. The results demonstrated an initial upregulation followed by a subsequent downregulation in the expression levels of glycolysis genes, peaking at 10 h and declining steadily thereafter. By contrast, the expression levels of TCA cycle genes exhibit an initial decrease followed by an increase, reaching their lowest point at 15 or 20 h and achieving their highest level at 30 h. These findings suggested that activation of the TCA cycle occurs during later stages of fermentation as a defensive response to elevated L-lactate concentrations and sodium lactate-induced stress.

In conclusion, the reduced expression levels of glycolytic genes and the elevated expression levels of TCA cycle genes in *H. coagulans* DSM1 during the late stage of fermentation are not conducive to L-LA production, which may directly contribute to the low yield of L-LA fermentation when NaOH is employed as a buffer.

### 3.7 Time-resolved expression analysis of transcriptional factors (TFs)

**Figure 7:**
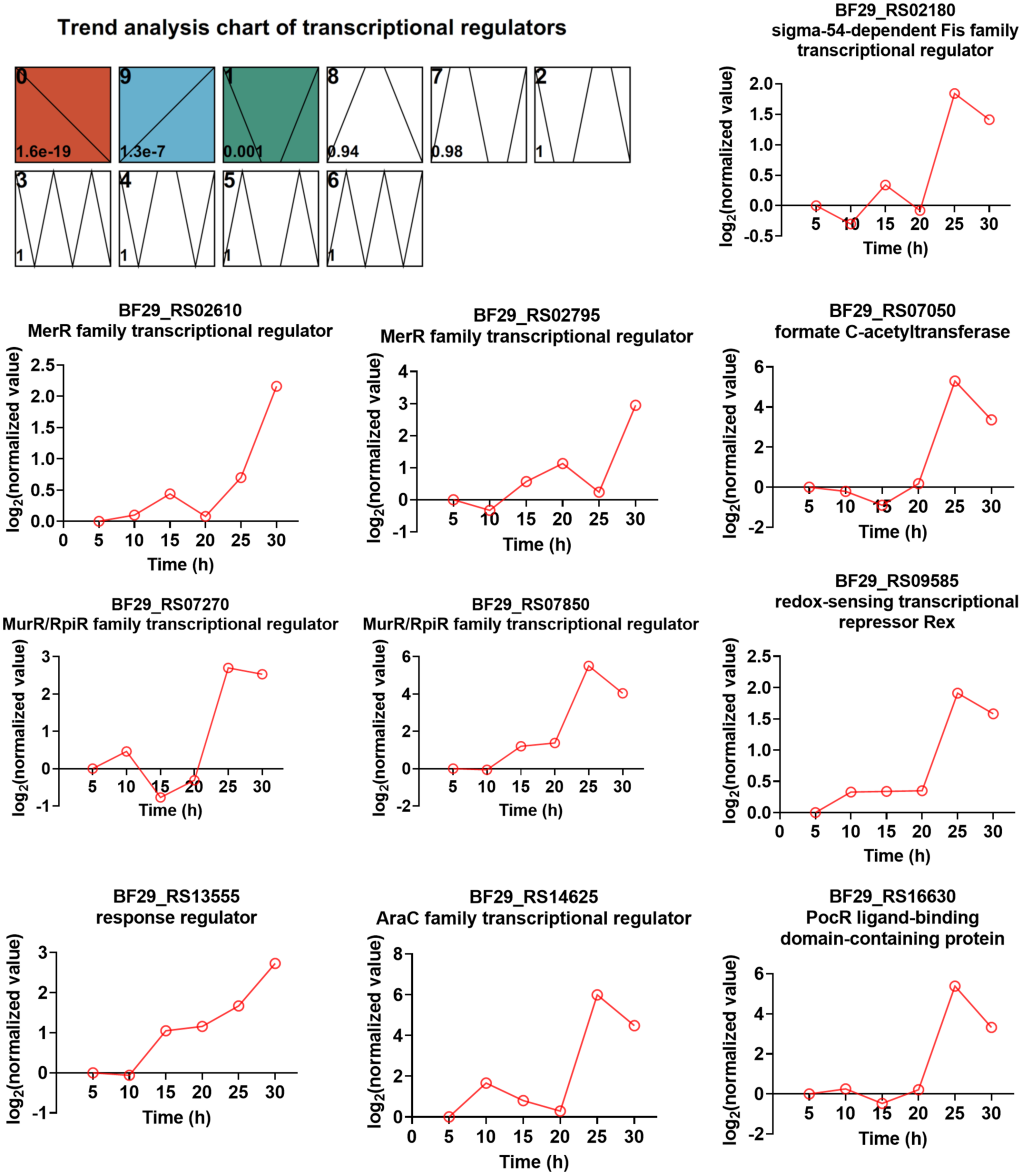
Time-series trend analysis of TFs by STEM and the TFs exhibited both an increasing trend during fermentation and ≥ 2-fold increased gene expression levels (RNA_30 h vs. RNA_20 h).

TFs are a group of protein molecules that specifically bind to upstream sequences of a gene’s 5′-end, ensuring precise spatiotemporal expression intensity. To investigate the involvement of TFs in L-LA production and strain resistance to lactate stress, we initially predicted all TFs encoded by the *H. coagulans* DSM1 genome using the P2TF database^1^ (Ortet et al., 2012), resulting the identification of 205 putative TFs. By employing the STEM method for time-series analysis, we observed significant enrichment in three modules (0, 9, and 1), with module 9 exhibiting an upward trend throughout the fermentation cycle encompassing a total of 39 TFs (Supplementary Table S10). Further screening revealed that among these 205 TFs, there were 33 TFs whose gene expression levels were more than twofold higher at 30 h compared with that at 20 h (Supplementary Table S11). The integration of information from Supplementary Tables S10, S11 enabled us to identify 10 TFs that consistently exhibited upregulation during fermentation, with significantly increased expression levels observed in the late stages. The expression trends of these TFs are illustrated in Figure 7.

Among these TFs, two belong to the MerR family of TFs (BF29_RS02610 and BF29_RS02795). The majority of MerR family regulators respond to various environmental stimuli, such as oxidative stress, heavy metals, or antibiotics (Brown et al., 2003). BF29_RS02610 shares a protein sequence identity of 64.1% with the HTH-type transcriptional regulator TnrA (Q45666) from *B. subtilis* 168, which functions as a master regulator of nitrogen assimilation primarily serving as an activator (Wray et al., 1996; He et al., 2023). BF29_RS02795 exhibits a protein sequence identity of 54.9% with the HTH-type transcriptional regulator GlnR (P37582) from *B. subtilis* 168, which acts as a repressor during nitrogen excess in nitrogen assimilation regulation (He et al., 2023). Interestingly, excessive nitrogen is not expected during mid- and late fermentation stages. Therefore, the regulatory mechanism of TnrA and GlnR in nitrogen uptake may not be entirely consistent between *H. coagulans* and *B. subtilis*. Two other TFS are classified as MurR/RpiR family TFs (BF29_RS07270 and BF29_RS07850), with their functions currently unknown. However, we observed that the homolog of BF29_RS07850 in *H. coagulans* 2–6 was significantly upregulated under lactate stress, suggesting its potential involvement in the lactate stress response (Qin et al., 2015). Another identified TF is BF29_RS13555, which functions as the response regulator CitT in a two-component system known as CitST. Previous studies conducted in *B. subtilis* have demonstrated that the sensor kinase CitS (BF29_RS13550 in DSM1) detects citrate present in the external environment and transmits a signal to activate CitT, thereby inducing the expression of CitM (BF29_RS13560 in DSM1), which serves as the primary transporter for citrate uptake (Repizo et al., 2006). Lastly, although not strictly categorized as a pure TF, BF29_RS07050 encodes formate C-acetyltransferase PflB, which is part of functional pyruvate formate lyase. The other part is the activating enzyme PflA encoded by BF29_RS07055. Pyruvate formate lyase is a crucial enzyme for mixed acid fermentation (Al-Bayati et al., 2017). The high expression levels of PflA and PflB suggest a shift in microbial metabolism from homolactic to mixed acid fermentation. Further exploration is required to elucidate the functional mechanisms underlying several other TFs involved in L-LA fermentation.

### 3.8 Time-resolved expression analysis of ABC transporters

**Figure 8:**
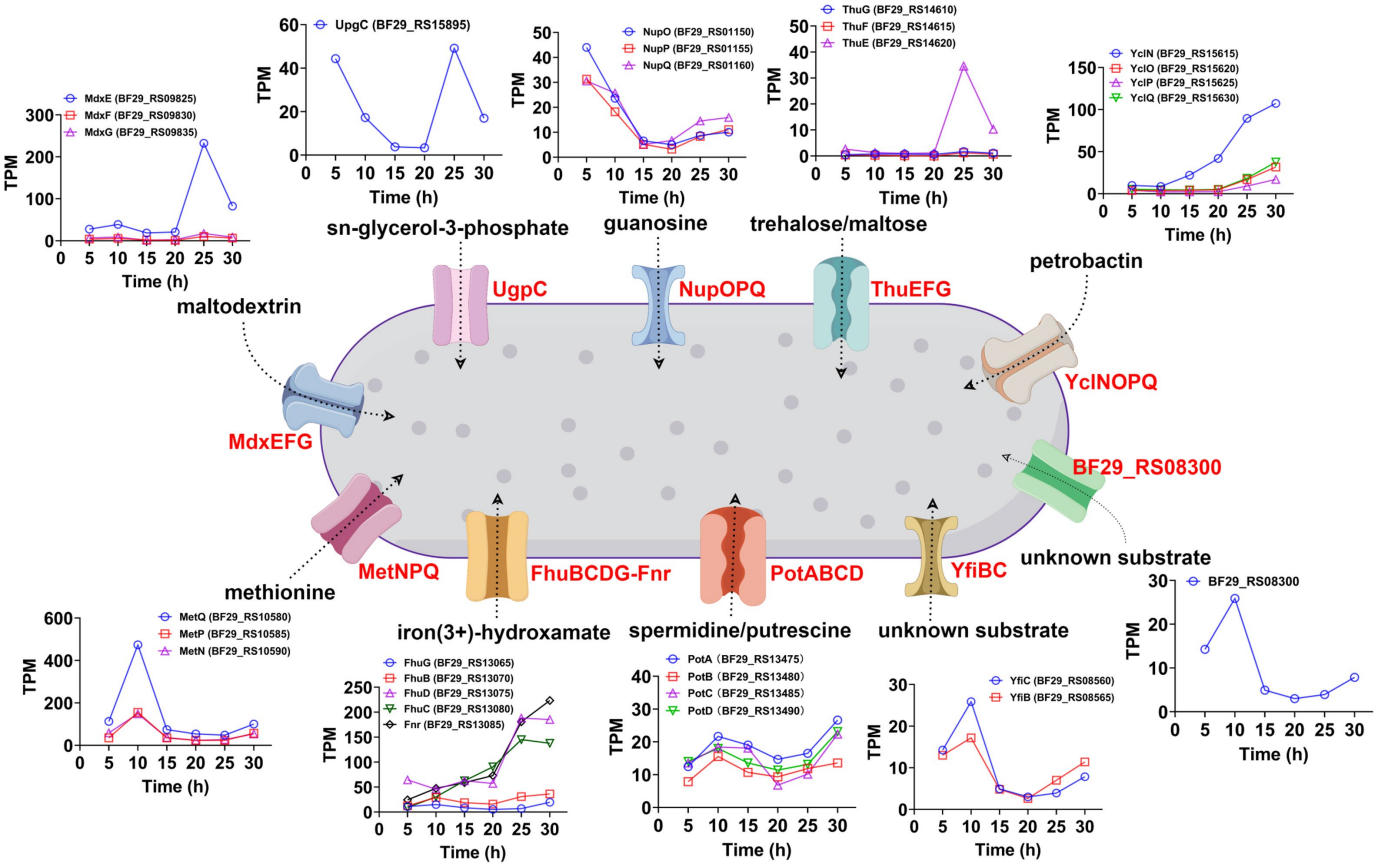
Temporal expression patterns and potential transport substrates of the significantly upregulated ABC transporters (30 h vs. 20 h) and other genes within their respective operons.

ATP-binding cassette (ABC) transporters are a widely distributed superfamily of integral membrane proteins that facilitate the ATP-dependent translocation of various substrates across membranes. In the KEGG enrichment analysis of DEGs between samples collected at 25 and 20 h, as well as samples collected at 30 and 25 h, ABC transporters exhibited significant enrichment (Supplementary Table S3). Therefore, we aimed to investigate the dynamic changes in all ABC transporters throughout the entire fermentation cycle. A total of 97 genes were annotated as ABC transporters in the genome of *H. coagulans* DSM1. Among them, 30 ABC transporters exhibited significant differences between the samples collected at 25 and 20 h, with 13 showing significantly increased expression and 17 displaying significantly decreased expression (Supplementary Table S12). Additionally, 20 ABC transporters showed significant differences between the samples collected at 30 and 25 h, including 8 with significantly increased expression and 12 with significantly decreased expression (Supplementary Table S13). Furthermore, when extending the time interval to 10 h, a total of 25 ABC transporters exhibited significant differences between the samples collected at 30 and 20 h. Among them, 16 transporters showed significantly increased expression and 9 displayed significantly decreased expression (Supplementary Table S14). These highly expressed ABC transporters belonged to 10 operons. The temporal expression patterns of these ABC transporters and other genes within their respective operons are depicted in Figure 8. Additionally, we conducted BLAST analysis with the UniProtKB/Swiss-Prot (swissprot) database to predict their potential transport substrates (Figure 8). Among the ABC transport systems, we further screened those exhibiting low expression levels during the early stage of fermentation but displaying significant upregulation after 20 h. These transport systems included YclNOPQ, FhuBCDG-Fnr, ThuEFG, and MdxEFG.

Among these systems, YclNOPQ represents an iron–siderophore transport system, with YclN (BF29_RS15615) and YclO (BF29_RS15620) acting as permease proteins, YclP (BF29_RS15625) serving as the ATP-binding protein, and YclQ (BF29_RS15630) functioning as the substrate-binding protein. The YclNOPQ complex is responsible for the uptake of petrobactin, which is a photoreactive catecholate siderophore produced by several members within the *Bacillus* group under limited iron availability (Zawadzka et al., 2009; Lee et al., 2011). Therefore, petrobactin may play a pivotal role in iron acquisition during L-LA fermentation by *H. coagulans* DSM1. FhuBCDG-Fnr is an additional ABC transport system, which is associated with the acquisition of metal ions (Pramanik and Braun, 2006). It encodes a system for importing iron(3+)-hydroxamate, consisting of five proteins: FhuG (BF29_RS13065), FhuB (BF29_RS13070), FhuD (BF29_RS13075), FhuC (BF29_RS13080), and Fnr (BF29_RS13085). Among these proteins, FhuG and FhuB function as permeases, whereas FhuD acts as the substrate-binding protein and FhuC serves as the ATP-binding protein. Additionally, the role of Fnr in this system is that of ferredoxin/flavodoxin NADP^+^ reductase. During late fermentation, the gene expression levels of FhuD, FhuC and FnR significantly increased (Figure 8), possibly to improve the uptake of trivalent iron ions. In summary, the gene expression levels of YclNOPQ and FhuBCDG-Fnr exhibited a gradual increase throughout fermentation, particularly after 20 h, indicating the significance of iron ion acquisition in *H. coagulans* DSM1-mediated L-LA production.

ThuEFG constitutes a trehalose/maltose transport system; with ThuF (BF29_RS14615) and ThuG (BF29_RS14610) serve as permease proteins, and ThuE (BF29_RS14620) acts as the substrate-binding protein (Jensen et al., 2002). Trehalose is widely recognized as a universal stress molecule and osmoprotectant, safeguarding cell integrity against various environmental injuries and nutritional limitations due to its unique physical properties (Argüelles, 2000). As illustrated in Figure 8, the expression level of the substrate binding protein BF29_RS14620 exhibited the most significant increase after 20 h, which was attributed to the high concentration of sodium lactate in the fermentation broth. MdxE (BF29_RS09825), MdxF (BF29_RS09830), and MdxG (BF29_RS09835) collectively encode a maltodextrin transport system known as MdxEFG, where MdxE functions as the substrate-binding protein and the latter two act as permease proteins (Schönert et al., 2006). Similarly, we found a substantial increase in MdxE expression after 20 h of fermentation (Figure 8). Therefore, *H. coagulans* DSM1 could utilize trehalose and maltodextrin to counteract sodium lactate stress during late-stage L-LA fermentation.

### 3.9 Suggestions for strain improvement

Based on the omics analysis mentioned above, we propose potential strategies for enhancing the capacity of *H. coagulans* DSM1 to produce L-LA. Firstly, it is feasible to augment the expression level or activity of key enzymes involved in L-LA metabolism during late fermentation. As depicted in Figure 6, crucial enzymes such as lactate dehydrogenase are significantly downregulated in later stages of fermentation. If these levels or activities of key enzymes can be maintained through genetic engineering techniques, it may facilitate L-LA synthesis. Secondly, there is potential for improving the strain’s ability to uptake and synthesize compatible solutes. For instance, this study revealed a significant upregulation of certain transporters associated with well-known compatible solutes like trehalose, maltodextrin, and glycerol during the late fermentation phase. These findings present novel targets for metabolic engineering aimed at enhancing strain resilience against sodium lactate stress. Thirdly, identification and regulation of novel TFs could be achieved. We have observed a substantial increase in the expression levels of several TFs during the late stage of L-LA fermentation (Figure 7). Functional characterization of these factors through gene knockout and other methodologies is necessary to determine their impact on L-LA production. Manipulation of expression levels or introduction random mutations in these selected TFs holds potential for improving strain performance in L-LA production. Additionally, transporter engineering represents another avenue for exploration. In order to maximize production capacity, improvements can be made in substrate uptake and product efflux capabilities. Our study has identified several ABC transporters that exhibit significantly increased expression levels during the late stage of L-LA fermentation (Figure 8), with particular emphasis on those involved iron ion acquisition.

## 4 Conclusion

In this study, we performed time-resolved transcriptomic and proteomic analyses of *H. coagulans* DSM1 during NaOH-buffered L-LA production. The expression levels of the glycolytic genes initially increased and then subsequently decreased, whereas the TCA cycle genes exhibited an initial decrease followed by a subsequent increase throughout the fermentation process. Moreover, we identified clusters of genes consisting of TFs and ABC transporters that demonstrated a progressive elevation of expression levels throughout the fermentation process, with significant upregulation observed at later stages. This investigation yields valuable insights into the response mechanisms of *H. coagulans* during NaOH-buffered L-LA fermentation and presents potential targets for metabolic engineering.

## Data availability statement

The datasets presented in this study can be found in online repositories. The names of the repository/repositories and accession number(s) can be found at: https://www.ncbi.nlm.nih.gov/genbank/, SRR25442713; https://www.ncbi.nlm.nih.gov/genbank/, SRR25442712; https://www.ncbi.nlm.nih.gov/genbank/, SRR25442711; https://www.ncbi.nlm.nih.gov/genbank/, SRR25442710; https://www.ncbi.nlm.nih.gov/genbank/, SRR25442709; https://www.ncbi.nlm.nih.gov/genbank/, SRR25442708.

## Author contributions

XH: Investigation, Writing – original draft. WT: Investigation, Writing – original draft. XW: Supervision, Writing – review & editing. JQ: Conceptualization, Funding acquisition, Resources, Supervision, Writing – review & editing.

## Footnotes

^1^ http://www.p2tf.org/
