# Supplementary material for: Time-resolved transcriptomic and proteomic profiling of Heyndrickxia coagulans during NaOH-buffered L-lactic acid production
Source: Front Microbiol. 2023 Nov 29;14:1296692. doi: 10.3389/fmicb.2023.1296692 (PMC10716427; doi:10.3389/fmicb.2023.1296692)
Supplement: Supplementary file 1 [file Data_Sheet_1.docx]

Supplementary Material

**Time-resolved** **transcriptomic and** **proteomic profiling of *Heyndrickxia coagulans* during NaOH-buffered L-lactic acid production**

**Xing Huang^†^, Wenzhe Tian^†^, Xiuwen Wang, Jiayang Qin^*^**

College of Pharmacy, Binzhou Medical University, Yantai 264003, China

**^†^**These authors share first authorship.

*Corresponding author

Address: College of Pharmacy, Binzhou Medical University, Yantai 264003, China

Phone: +86-535-6913254; Fax: +86-535-6913718

Email addresses:

XH: [f007x@sina.com](mailto:f007x@sina.com); WT: [1158807207@qq.com](mailto:1158807207@qq.com);

XW: [wangxiuwen0822@163.com](mailto:wangxiuwen0822@163.com); JQ: [qinjy@bzmc.edu.cn](mailto:qinjy@bzmc.edu.cn)

**Table S1** Primers for qRT-PCR analysis with target gene information.

| Locus tag | Description | Primers (5΄→3΄) | Length |
| --- | --- | --- | --- |
| BF29_RS02315 | LysR family transcriptional regulator | TCAGTAGAAGCAGGATTAGG  ATTTACGTGAGAAGGGAGTG | 115 bp |
| BF29_RS07850 | MurR/RpiR family transcriptional regulator | GCATTCATTCCCGTGTCTTC  CTGGTTGATCGTGCTGTGG | 124 bp |
| BF29_RS13920 | Helix-turn-helix domain-containing protein | TTCCAAACAGAATGGCTCA  CTCCTCAATCCGTAAACAA | 182 bp |
| BF29_RS14265 | Hypothetical protein | TCCCGATTCAGCAGATTTAG  AATCATCGTCCCTTTGTCCC | 82 bp |
| BF29_RS11110 | 16S rRNA | AGGCTGAAACTCAAAGGAAT  AACCCAACATCTCACGACAC | 193 bp |

**Table S2** Comparison of gene expression between RNA-seq and qRT-PCR.

| Locus tag | Description | Fold change (20 h vs 5 h) | |
| --- | --- | --- | --- |
|  |  | RNA-seq | qRT-PCR |
| BF29_RS02315 | LysR family transcriptional regulator | 4.33 | 5.28 |
| BF29_RS07850 | MurR/RpiR family transcriptional regulator | 2.81 | 41.9 |
| BF29_RS13920 | Helix-turn-helix domain-containing protein | 4.18 | 3.63 |
| BF29_RS14265 | Hypothetical protein | 6.16 | 17.8 |

**Table S3** Significantly enriched KEGG pathways between multiple time points in transcriptomic analysis.

| Group | Pathway ID | Pathway Description | Gene Num | Ratio in study | Ratio in pop | *P*_adjust_ |
| --- | --- | --- | --- | --- | --- | --- |
| 20 h vs.  15 h | map00290 | Valine, leucine and isoleucine biosynthesis | 6 | 6/153 | 12/1806 | 0.014 |
| 25 h vs.  20 h | map00020 | Citrate cycle (TCA cycle) | 17 | 17/263 | 22/1806 | 0.000 |
|  | map02060 | Phosphotransferase system (PTS) | 13 | 13/263 | 18/1806 | 0.000 |
|  | map00071 | Fatty acid degradation | 11 | 11/263 | 16/1806 | 0.000 |
|  | map02010 | ABC transporters | 29 | 29/263 | 85/1806 | 0.000 |
|  | map00720 | Carbon fixation pathways in prokaryotes | 12 | 12/263 | 24/1806 | 0.001 |
|  | map00040 | Pentose and glucuronate interconversions | 7 | 7/263 | 10/1806 | 0.002 |
|  | map00650 | Butanoate metabolism | 9 | 9/263 | 18/1806 | 0.005 |
|  | map03320 | PPAR signaling pathway | 5 | 5/263 | 6/1806 | 0.005 |
|  | map00640 | Propanoate metabolism | 12 | 12/263 | 30/1806 | 0.007 |
|  | map00630 | Glyoxylate and dicarboxylate metabolism | 11 | 11/263 | 28/1806 | 0.012 |
|  | map00561 | Glycerolipid metabolism | 8 | 8/263 | 17/1806 | 0.014 |
|  | map04146 | Peroxisome | 5 | 5/263 | 8/1806 | 0.023 |
|  | map04920 | Adipocytokine signaling pathway | 3 | 3/263 | 3/1806 | 0.026 |
|  | map04216 | Ferroptosis | 3 | 3/263 | 3/1806 | 0.026 |
|  | map00281 | Geraniol degradation | 3 | 3/263 | 3/1806 | 0.026 |
|  | map00500 | Starch and sucrose metabolism | 11 | 11/263 | 32/1806 | 0.027 |
|  | map00520 | Amino sugar and nucleotide sugar metabolism | 11 | 11/263 | 32/1806 | 0.027 |
|  | map00280 | Valine, leucine and isoleucine degradation | 8 | 8/263 | 20/1806 | 0.029 |
|  | map00620 | Pyruvate metabolism | 12 | 12/263 | 39/1806 | 0.042 |
| 30 h vs.  25 h | map00330 | Arginine and proline metabolism | 8 | 8/195 | 17/1806 | 0.0082 |
|  | map02010 | ABC transporters | 21 | 21/195 | 85/1806 | 0.0145 |
|  | map00791 | Atrazine degradation | 3 | 3/195 | 3/1806 | 0.041 |
|  | map02060 | Phosphotransferase system (PTS) | 7 | 7/195 | 18/1806 | 0.0429 |
| 30 h vs.  20 h | map00020 | Citrate cycle (TCA cycle) | 17 | 17/342 | 22/1806 | 0 |
|  | map00071 | Fatty acid degradation | 11 | 11/342 | 16/1806 | 0.001 |
|  | map00650 | Butanoate metabolism | 10 | 10/342 | 18/1806 | 0.0166 |
|  | map00720 | Carbon fixation pathways in prokaryotes | 12 | 12/342 | 24/1806 | 0.0218 |
|  | map00230 | Purine metabolism | 18 | 18/342 | 47/1806 | 0.0273 |
|  | map03320 | PPAR signaling pathway | 5 | 5/342 | 6/1806 | 0.0296 |

**Table S4** Top 20 genes exhibiting significantly increased expression levels in the 30 h samples compared with the 20 h samples.

| Locus tag | Description | Log_2_FC | *P*_adjust_ |
| --- | --- | --- | --- |
| BF29_RS04425 | transposase family protein | 9.78 | 0.00 |
| BF29_RS09895 | transposase family protein | 8.03 | 0.00 |
| BF29_RS12685 | transposase family protein | 7.35 | 0.00 |
| BF29_RS13625 | aquaporin family protein | 7.12 | 0.00 |
| BF29_RS16355 | ISLre2 family transposase | 6.81 | 0.00 |
| BF29_RS02245 | IS21 family transposase | 6.08 | 0.00 |
| BF29_RS16635 | acetate kinase | 4.63 | 0.00 |
| BF29_RS06670 | glycoside-pentoside-hexuronide (GPH):cation symporter | 4.28 | 0.00 |
| BF29_RS16105 | MFS transporter | 4.06 | 0.01 |
| BF29_RS17730 | acetyl-CoA acetyltransferase | 4.06 | 0.01 |
| BF29_RS14675 | SDR family NAD(P)-dependent oxidoreductase | 4.00 | 0.00 |
| BF29_RS14625 | AraC family transcriptional regulator | 3.97 | 0.00 |
| BF29_RS14480 | L-lactate permease | 3.88 | 0.00 |
| BF29_RS04960 | electron transfer flavoprotein subunit alpha/FixB family protein | 3.72 | 0.00 |
| BF29_RS08590 | DUF202 domain-containing protein | 3.70 | 0.00 |
| BF29_RS03720 | ComGF family competence protein | 3.70 | 0.02 |
| BF29_RS14615 | sugar ABC transporter permease | 3.70 | 0.02 |
| BF29_RS13630 | glycerol kinase GlpK | 3.62 | 0.00 |
| BF29_RS13425 | spore coat protein | 3.59 | 0.00 |
| BF29_RS04980 | AMP-binding protein | 3.52 | 0.00 |

**Table S5** Top 10 proteins exhibiting a significant increase in protein expression in the 30 h samples compared with the 20 h samples.

| Locus tag | Description | Log_2_FC | log_10_P_adjust_ |
| --- | --- | --- | --- |
| BF29_RS01725 | MFS transporter | 3.89 | 0 |
| BF29_RS04995 | CvpA family protein | 3.05 | 0 |
| BF29_RS03835 | ABC transporter ATP-binding protein | 2.83 | 0 |
| BF29_RS07580 | TIGR02678 family protein | 2.20 | 0 |
| BF29_RS05155 | glyceraldehyde-3-phosphate dehydrogenase | 2.10 | 0 |
| BF29_RS11175 | tRNA1(Val) (adenine(37)-N6)-methyltransferase | 1.88 | 0 |
| BF29_RS06675 | glucuronate isomerase | 1.46 | 0 |
| BF29_RS14985 | short chain dehydrogenase family protein | 1.40 | 0 |
| BF29_RS06895 | ABC transporter permease | 1.26 | 0 |
| BF29_RS01605 | MBL fold metallo-hydrolase | 1.20 | 0 |

**Table S6** Significantly upregulated genes in the 30 h samples compared with the 20 h samples by both transcriptomic and proteomic analyses.

| Locus tag | Description | Log_2_FC (gene) | Log_2_FC (protein) |
| --- | --- | --- | --- |
| BF29_RS00045 | 2-oxo acid dehydrogenase subunit E2 | 3.41 | 0.31 |
| BF29_RS00250 | N-acetyltransferase | 1.56 | 0.36 |
| BF29_RS01590 | rhodanese-related sulfurtransferase | 2.16 | 0.33 |
| BF29_RS01960 | nitronate monooxygenase | 2.20 | 0.83 |
| BF29_RS02610 | MerR family transcriptional regulator | 1.85 | 0.37 |
| BF29_RS03335 | site-specific tyrosine recombinase XerD | 1.08 | 0.81 |
| BF29_RS04305 | Rrf2 family transcriptional regulator | 1.32 | 0.36 |
| BF29_RS04920 | succinate dehydrogenase iron-sulfur subunit | 1.84 | 0.32 |
| BF29_RS04965 | electron transfer flavoprotein subunit beta/FixA family protein | 2.89 | 0.28 |
| BF29_RS04970 | enoyl-CoA hydratase | 1.63 | 0.82 |
| BF29_RS05155 | glyceraldehyde-3-phosphate dehydrogenase | 1.14 | 2.10 |
| BF29_RS05490 | acetate--CoA ligase | 2.42 | 0.28 |
| BF29_RS06535 | 3-hydroxyacyl-CoA dehydrogenase/enoyl-CoA hydratase family protein | 2.16 | 0.66 |
| BF29_RS06675 | glucuronate isomerase | 3.05 | 1.46 |
| BF29_RS06680 | altronate dehydratase family protein | 2.46 | 0.39 |
| BF29_RS06695 | bifunctional 2-keto-4-hydroxyglutarate aldolase/2-keto-3-deoxy-6-phosphogluconate aldolase | 1.37 | 1.13 |
| BF29_RS07000 | amidase domain-containing protein | 1.48 | 0.86 |
| BF29_RS07050 | formate C-acetyltransferase | 2.98 | 0.81 |
| BF29_RS07270 | MurR/RpiR family transcriptional regulator | 2.62 | 0.67 |
| BF29_RS07275 | PTS transporter subunit EIIC | 2.55 | 0.40 |
| BF29_RS08290 | amino acid permease | 1.33 | 0.67 |
| BF29_RS08660 | deoxyribose-phosphate aldolase | 1.04 | 0.27 |
| BF29_RS08705 | DUF979 domain-containing protein | 1.22 | 0.46 |
| BF29_RS08710 | DUF969 domain-containing protein | 1.04 | 0.50 |
| BF29_RS09040 | isocitrate lyase | 2.94 | 0.66 |
| BF29_RS09585 | redox-sensing transcriptional repressor Rex | 1.02 | 0.56 |
| BF29_RS10255 | 4Fe-4S dicluster domain-containing protein | 1.32 | 0.29 |
| BF29_RS10285 | YwhD family protein | 1.02 | 0.29 |
| BF29_RS12560 | ArsR family transcriptional regulator | 1.06 | 0.58 |
| BF29_RS12635 | phage minor capsid protein | 1.50 | 0.27 |
| BF29_RS13520 | lactate utilization protein C | 1.21 | 0.38 |
| **BF29_RS13625** | **aquaporin family protein** | **7.12** | **0.94** |
| BF29_RS13635 | glycerol-3-phosphate dehydrogenase/oxidase | 1.93 | 0.56 |
| BF29_RS14215 | beta-galactosidase | 1.46 | 0.32 |
| BF29_RS14310 | carboxylating nicotinate-nucleotide diphosphorylase | 2.32 | 0.51 |
| BF29_RS14320 | IscS subfamily cysteine desulfurase | 2.51 | 0.29 |
| BF29_RS14820 | citrate synthase | 2.54 | 0.69 |
| BF29_RS14825 | 2-methylcitrate dehydratase | 2.92 | 0.39 |
| BF29_RS14935 | C40 family peptidase | 1.14 | 0.54 |
| BF29_RS16635 | acetate kinase | 4.63 | 0.29 |

**Table S7** Significantly downregulated genes in the 30 h samples compared with the 20 h samples by both transcriptomic and proteomic analyses.

| Locus tag | Description | Log_2_FC (gene) | Log_2_FC (protein) |
| --- | --- | --- | --- |
| BF29_RS00360 | peptidoglycan editing factor PgeF | -1.31 | -0.61 |
| BF29_RS02770 | DUF896 domain-containing protein | -1.11 | -0.99 |
| BF29_RS05045 | hypothetical protein | -1.43 | -0.34 |
| BF29_RS05080 | 7-cyano-7-deazaguanine synthase QueC | -1.19 | -0.27 |
| BF29_RS05330 | alpha/beta-type small acid-soluble spore protein | -1.47 | -0.80 |
| BF29_RS05385 | proline dehydrogenase family protein | -1.86 | -0.36 |
| BF29_RS08040 | nitrous oxide-stimulated promoter family protein | -1.34 | -0.46 |
| BF29_RS08045 | PTS glucose transporter subunit IIA | -1.43 | -0.67 |
| BF29_RS09410 | hypothetical protein | -1.32 | -0.31 |
| BF29_RS09920 | single-stranded DNA-binding protein | -1.23 | -0.41 |
| BF29_RS10400 | GNAT family N-acetyltransferase | -1.00 | -0.55 |
| **BF29_RS10675** | **ABC transporter ATP-binding protein** | **-7.36** | **-1.94** |
| BF29_RS11340 | serine/threonine protein kinase | -1.22 | -0.53 |
| BF29_RS13505 | hypothetical protein | -2.16 | -0.56 |
| BF29_RS14290 | YifB family Mg chelatase-like AAA ATPase | -1.16 | -0.55 |
| BF29_RS14470 | hypothetical protein | -1.14 | -0.72 |
| BF29_RS15670 | phosphate ABC transporter substrate-binding protein PstS family protein | -7.88 | -0.69 |
| BF29_RS16815 | DUF1232 domain-containing protein | -1.29 | -0.86 |
| BF29_RS17035 | helix-turn-helix transcriptional regulator | -1.28 | -0.30 |

**Table S8** Genes encompassed in module 49 during the time-series analysis of transcriptome and their corresponding expression trends.

| Locus tag | log_2_(normalized value of genes) | | | | | |
| --- | --- | --- | --- | --- | --- | --- |
|  | 5 h | 10 h | 15 h | 20 h | 25 h | 30 h |
| BF29_RS00155 | 0 | 0.63 | 1.58 | 2.2 | 3.2 | 2.42 |
| BF29_RS00250 | 0 | -0.87 | 0.1 | 0.33 | 1.56 | 1.9 |
| BF29_RS00525 | 0 | 1.4 | 3.23 | 2.99 | 3.34 | 3.31 |
| BF29_RS01460 | 0 | -0.16 | 0.82 | 1.45 | 1.78 | 1.53 |
| BF29_RS01470 | 0 | -0.68 | 1.05 | 1.62 | 1.75 | 3.17 |
| BF29_RS01605 | 0 | 0.52 | 1.01 | 1.22 | 1.41 | 1.25 |
| BF29_RS01615 | 0 | 2.3 | 1.89 | 1.73 | 3.44 | 3.66 |
| BF29_RS01640 | 0 | 0.33 | 0.9 | 1.11 | 1.44 | 1.07 |
| BF29_RS01840 | 0 | 0.3 | 0.75 | 0.56 | 1.46 | 1.58 |
| BF29_RS01845 | 0 | 0.37 | 0.74 | 0.71 | 0.82 | 1.46 |
| BF29_RS01885 | 0 | 1.1 | 0.82 | 1.63 | 1.19 | 1.85 |
| BF29_RS01940 | 0 | 0.63 | 1.63 | 2.76 | 2.38 | 3.38 |
| BF29_RS02190 | 0 | 1.01 | 1.19 | 1.53 | 1.45 | 1.67 |
| BF29_RS02210 | 0 | 0.33 | 0.87 | 1.21 | 1.11 | 1.28 |
| BF29_RS02525 | 0 | 0.84 | 2.29 | 2.32 | 7.17 | 6.18 |
| BF29_RS02530 | 0 | 0.58 | 0.71 | 1.42 | 6.37 | 4.91 |
| BF29_RS02555 | 0 | 0.01 | 0.3 | 0.68 | 0.82 | 1.03 |
| BF29_RS02610 | 0 | 0.19 | 0.62 | 0.41 | 1.01 | 2.32 |
| BF29_RS02625 | 0 | 1.32 | 1.28 | 2.43 | 2.78 | 3.23 |
| BF29_RS02735 | 0 | -0.07 | 0.8 | 1.33 | 2.53 | 2.32 |
| BF29_RS03705 | 0 | 0.98 | 1.55 | 1.49 | 1.66 | 1.74 |
| BF29_RS03755 | 0 | -0.12 | 0.44 | 0.98 | 1.5 | 1.71 |
| BF29_RS03880 | 0 | 0.2 | 2.41 | 2.89 | 4.52 | 4.83 |
| BF29_RS04000 | 0 | 0.51 | 0.42 | 0.79 | 0.85 | 1.15 |
| BF29_RS04010 | 0 | 0.23 | 0.16 | 0.64 | 1.18 | 1.48 |
| BF29_RS04120 | 0 | -0.45 | -0.06 | 1.3 | 1.83 | 2.13 |
| BF29_RS04375 | 0 | 0.52 | 0.78 | 1.24 | 1.45 | 1.4 |
| BF29_RS04980 | 0 | -0.62 | 0.69 | 0.69 | 5.83 | 4.16 |
| BF29_RS05150 | 0 | 0.06 | 0.38 | 2.12 | 2.7 | 4.29 |
| BF29_RS05155 | 0 | -0.51 | 1.41 | 3.57 | 3.85 | 4.7 |
| BF29_RS05245 | 0 | 2.16 | 1.97 | 3 | 2.7 | 3.1 |
| BF29_RS05285 | 0 | 0.21 | 0.79 | 0.6 | 0.95 | 1.07 |
| BF29_RS05410 | 0 | -0.24 | 0.78 | 0.73 | 0.6 | 1.13 |
| BF29_RS05465 | 0 | 0.49 | 0.84 | 0.97 | 0.95 | 1.02 |
| BF29_RS05505 | 0 | 1.21 | 1.22 | 1.17 | 2.55 | 1.92 |
| BF29_RS05760 | 0 | 0.5 | 0.54 | 0.8 | 1.28 | 1.69 |
| BF29_RS05775 | 0 | -0.31 | 0.35 | 0.99 | 1.05 | 1.11 |
| BF29_RS06060 | 0 | -0.57 | 0.09 | 0.88 | 1.24 | 1.46 |
| BF29_RS06190 | 0 | 1.78 | 1.87 | 1.76 | 2.37 | 2.36 |
| BF29_RS06655 | 0 | 0.26 | 1.99 | 2.86 | 2.63 | 2.79 |
| BF29_RS06670 | 0 | 1.16 | 1.55 | 2.22 | 6.82 | 6.58 |
| BF29_RS06675 | 0 | 0 | 0.65 | 2.24 | 5.3 | 5.3 |
| BF29_RS06680 | 0 | 0.27 | 0.58 | 2.07 | 4.68 | 4.57 |
| BF29_RS06685 | 0 | 0.26 | 1.42 | 2.61 | 4.47 | 4.29 |
| BF29_RS06690 | 0 | 0.23 | 0.86 | 1.95 | 3.5 | 4 |
| BF29_RS06695 | 0 | 0.28 | 1.08 | 2.53 | 3.98 | 4.04 |
| BF29_RS06870 | 0 | 1.01 | 0.76 | 1.59 | 4.18 | 3.17 |
| BF29_RS07110 | 0 | -0.04 | 0.87 | 0.49 | 0.87 | 1.79 |
| BF29_RS07215 | 0 | 1.06 | 1.36 | 1.93 | 2.98 | 2.32 |
| BF29_RS07280 | 0 | 0.29 | 0.28 | 0.72 | 4.23 | 3.32 |
| BF29_RS07285 | 0 | 0.02 | 1.54 | 2.12 | 3.71 | 2.68 |
| BF29_RS07345 | 0 | -0.04 | 0.77 | 0.85 | 1.21 | 1.08 |
| BF29_RS07365 | 0 | 1.05 | 1.94 | 2.11 | 2.31 | 2.06 |
| BF29_RS07405 | 0 | -0.38 | 0.14 | 0.42 | 0.35 | 0.94 |
| BF29_RS07500 | 0 | 0.84 | 1.97 | 2 | 2.12 | 2.93 |
| BF29_RS07520 | 0 | 1.64 | 2.67 | 2.45 | 2.74 | 3.12 |
| BF29_RS07565 | 0 | 0.15 | 0.18 | 1.89 | 5.59 | 4.92 |
| BF29_RS07850 | 0 | 0.43 | 1.38 | 2 | 6.1 | 4.44 |
| BF29_RS07890 | 0 | 0.69 | 1.19 | 1.01 | 1.09 | 1.72 |
| BF29_RS07900 | 0 | 0.42 | 0.78 | 0.49 | 1.32 | 1.34 |
| BF29_RS08085 | 0 | -0.25 | 2.58 | 2.9 | 3.14 | 3.56 |
| BF29_RS08090 | 0 | -2.19 | 0.84 | 2.17 | 1.81 | 2.63 |
| BF29_RS08115 | 0 | -0.55 | 1.72 | 1.16 | 3.44 | 2.86 |
| BF29_RS08160 | 0 | 0.05 | 0.46 | 0.77 | 0.93 | 1.31 |
| BF29_RS08165 | 0 | 0.57 | 1.1 | 1.13 | 1.37 | 1.42 |
| BF29_RS08170 | 0 | 0.26 | 1.63 | 1.84 | 2.32 | 1.8 |
| BF29_RS08290 | 0 | -0.84 | 0.14 | 0.32 | 1.12 | 1.79 |
| BF29_RS08470 | 0 | 1.38 | 2.67 | 3.16 | 3.15 | 3.19 |
| BF29_RS08545 | 0 | 3.3 | 3.14 | 3.59 | 5.58 | 5.02 |
| BF29_RS08610 | 0 | 0.48 | 0.77 | 0.92 | 0.86 | 1.13 |
| BF29_RS08655 | 0 | 0.7 | 0.49 | 0.89 | 2.61 | 1.87 |
| BF29_RS08660 | 0 | 0.6 | 0.47 | 0.68 | 2.7 | 1.76 |
| BF29_RS08665 | 0 | 0.61 | 0.65 | 0.93 | 2.97 | 1.91 |
| BF29_RS08670 | 0 | 0.53 | 0.93 | 1.07 | 3.12 | 1.86 |
| BF29_RS09020 | 0 | 1.64 | 2.39 | 2.74 | 3.19 | 2.96 |
| BF29_RS09095 | 0 | 0.46 | 0.72 | 0.79 | 1.99 | 1.49 |
| BF29_RS09325 | 0 | 0.75 | 2.1 | 2.2 | 2.4 | 2.65 |
| BF29_RS09330 | 0 | -0.09 | 1.04 | 1 | 1.12 | 1.39 |
| BF29_RS09465 | 0 | 0.01 | 0.35 | 1.68 | 2.07 | 1.81 |
| BF29_RS09580 | 0 | -0.15 | 0.87 | 1.28 | 2.08 | 1.97 |
| BF29_RS09585 | 0 | 0.27 | 0.34 | 0.48 | 2 | 1.59 |
| BF29_RS10145 | 0 | 0.5 | 0.29 | 0.88 | 0.72 | 1.27 |
| BF29_RS10235 | 0 | 0.84 | 2.08 | 1.42 | 2.99 | 3.2 |
| BF29_RS10240 | 0 | 0.98 | 1.65 | 1.26 | 2.7 | 2.69 |
| BF29_RS10250 | 0 | 0.7 | 1.21 | 1.35 | 2.41 | 2.35 |
| BF29_RS10355 | 0 | 0.41 | 0.96 | 0.99 | 1.04 | 1.09 |
| BF29_RS10375 | 0 | 0.84 | 1.55 | 1.59 | 1.7 | 2.25 |
| BF29_RS10520 | 0 | 0.76 | 2.37 | 2.81 | 4.31 | 3.58 |
| BF29_RS10525 | 0 | 1.1 | 3.02 | 3.18 | 4.42 | 3.66 |
| BF29_RS10530 | 0 | 0.16 | 1.76 | 1.5 | 2.8 | 2.61 |
| BF29_RS10535 | 0 | -0.22 | 1.26 | 0.99 | 2.06 | 2.29 |
| BF29_RS10760 | 0 | 0.75 | 1.97 | 2.75 | 2.72 | 2.74 |
| BF29_RS10765 | 0 | 1.27 | 2.01 | 2.75 | 2.58 | 3.06 |
| BF29_RS10965 | 0 | 0.57 | 1.99 | 1.74 | 2.19 | 2.04 |
| BF29_RS10970 | 0 | 0.48 | 2.4 | 2.39 | 2.76 | 2.6 |
| BF29_RS10975 | 0 | 0.96 | 3.31 | 3.03 | 3.51 | 3.4 |
| BF29_RS11590 | 0 | 0.7 | 0.84 | 1.24 | 1.18 | 1.36 |
| BF29_RS11595 | 0 | 1.14 | 1.52 | 1.56 | 1.74 | 1.95 |
| BF29_RS11605 | 0 | 0.45 | 1.43 | 1.49 | 1.73 | 2.07 |
| BF29_RS12020 | 0 | 1.03 | 2.2 | 1.92 | 3.24 | 3.1 |
| BF29_RS12030 | 0 | 0.75 | 1.75 | 1.81 | 3.07 | 2.2 |
| BF29_RS12035 | 0 | 1.17 | 1.27 | 1 | 2.13 | 1.99 |
| BF29_RS13075 | 0 | -0.61 | -0.03 | -0.04 | 1.57 | 1.4 |
| BF29_RS13080 | 0 | 1.63 | 2.79 | 3.4 | 4.01 | 3.8 |
| BF29_RS13085 | 0 | 0.86 | 1.33 | 1.72 | 2.99 | 3.14 |
| BF29_RS13135 | 0 | 0.53 | 1.08 | 0.96 | 1.05 | 1.42 |
| BF29_RS13545 | 0 | 1.08 | 3.82 | 4.61 | 3.71 | 6.22 |
| BF29_RS13550 | 0 | -0.03 | 1.69 | 1.8 | 1.62 | 2.43 |
| BF29_RS13555 | 0 | -0.21 | 1.1 | 1.34 | 1.74 | 2.64 |
| BF29_RS13560 | 0 | 0.56 | 1.5 | 1.24 | 2.03 | 2.67 |
| BF29_RS13665 | 0 | 1.65 | 4.25 | 5.38 | 5.47 | 5.05 |
| BF29_RS13715 | 0 | 0.49 | 1.19 | 2.09 | 5.07 | 4.47 |
| BF29_RS13765 | 0 | 0.41 | 1.04 | 1.16 | 1.41 | 1.89 |
| BF29_RS13870 | 0 | 1.24 | 1.97 | 2.15 | 2.19 | 2.89 |
| BF29_RS13930 | 0 | -0.1 | 0.55 | 0.65 | 3.47 | 2.92 |
| BF29_RS14010 | 0 | -0.46 | 0.23 | 0.15 | 0.47 | 0.84 |
| BF29_RS14025 | 0 | -0.22 | -0.06 | 0.77 | 0.82 | 0.77 |
| BF29_RS14045 | 0 | 0.6 | 4.77 | 5.29 | 5.57 | 5.29 |
| BF29_RS14050 | 0 | 0.59 | 4.75 | 5.32 | 5.73 | 5.83 |
| BF29_RS14320 | 0 | 1.81 | 1.89 | 2.51 | 4.84 | 5.15 |
| BF29_RS14410 | 0 | 0.07 | 1.68 | 1.73 | 2.18 | 1.83 |
| BF29_RS14565 | 0 | -0.01 | 0.58 | 0.45 | 0.96 | 1.14 |
| BF29_RS14625 | 0 | 1.61 | 1.14 | 1.17 | 5.85 | 4.2 |
| BF29_RS14750 | 0 | 2.93 | 1.55 | 2.81 | 4.79 | 4.68 |
| BF29_RS14760 | 0 | 0.75 | 0.74 | 1.12 | 1.24 | 1.16 |
| BF29_RS14815 | 0 | 0.04 | 0.64 | 1 | 1.27 | 0.84 |
| BF29_RS14865 | 0 | 0.61 | 3.06 | 2.99 | 2.86 | 3.67 |
| BF29_RS14945 | 0 | 0.1 | 0.18 | 1.07 | 1.37 | 2.09 |
| BF29_RS15140 | 0 | -0.76 | -0.12 | 0.8 | 1.08 | 1.34 |
| BF29_RS15145 | 0 | -0.91 | 0.54 | 1.24 | 1.11 | 1.55 |
| BF29_RS15165 | 0 | 0.69 | 3.26 | 3.25 | 3.93 | 3.45 |
| BF29_RS15365 | 0 | -0.32 | 1.7 | 1.48 | 1.84 | 1.66 |
| BF29_RS15615 | 0 | -0.3 | 1.21 | 2.17 | 3.19 | 3.37 |
| BF29_RS15620 | 0 | -0.34 | -0.12 | 0.31 | 1.95 | 2.77 |
| BF29_RS15630 | 0 | -0.26 | -0.1 | 0.29 | 1.86 | 2.73 |
| BF29_RS15635 | 0 | 0.58 | 0.58 | 0.56 | 0.63 | 1.11 |
| BF29_RS15950 | 0 | -0.35 | 0.2 | 0.9 | 2.86 | 1.74 |
| BF29_RS16015 | 0 | 1.05 | 1.63 | 1.97 | 2.33 | 2.01 |
| BF29_RS16070 | 0 | -0.3 | 0.75 | 1.29 | 1.03 | 1.47 |
| BF29_RS16095 | 0 | 0.55 | 0.24 | 1.66 | 2.48 | 2.43 |
| BF29_RS16100 | 0 | 0.62 | -0.03 | 1.71 | 2.26 | 2.44 |
| BF29_RS16165 | 0 | 1.05 | 2.15 | 2.27 | 2.83 | 2.35 |
| BF29_RS16170 | 0 | 1.44 | 2.25 | 2.58 | 2.95 | 2.63 |
| BF29_RS16265 | 0 | -0.26 | 0.4 | 0.4 | 0.96 | 0.77 |
| BF29_RS16395 | 0 | 0.15 | 1.65 | 1.53 | 1.81 | 1.5 |
| BF29_RS16415 | 0 | 0.06 | 0.22 | 0.13 | 0.4 | 1.14 |
| BF29_RS16520 | 0 | 2.14 | 3.39 | 4.02 | 4.17 | 4.19 |
| BF29_RS16600 | 0 | 2.82 | 2.69 | 2.93 | 5.46 | 5.3 |
| BF29_RS16635 | 0 | 2.76 | 1.66 | 2.11 | 6.82 | 6.76 |
| BF29_RS16770 | 0 | -0.12 | 0.26 | 0.76 | 1.31 | 0.8 |
| BF29_RS16830 | 0 | 0.39 | 0.72 | 1.53 | 1.61 | 2.31 |
| BF29_RS17000 | 0 | 0.7 | 1.65 | 1.73 | 2.76 | 2.59 |
| BF29_RS17070 | 0 | 3.05 | 3.52 | 4.64 | 4.81 | 4.83 |
| Novel00013 | 0 | 0.09 | 1 | 1.46 | 1.69 | 1.25 |
| Novel00049 | 0 | 1.1 | 1.22 | 1.17 | 1.54 | 1.54 |
| Novel00086 | 0 | 0.33 | 1.99 | 3.07 | 4.85 | 4.06 |
| sRNA00060 | 0 | 0.12 | 0.31 | 0.9 | 1.71 | 1.12 |

**Table S9** Proteins encompassed in module 49 during the time-series analysis of proteome and their corresponding expression trends.

| Locus tag | log_2_(normalized value of proteins) | | | | | |
| --- | --- | --- | --- | --- | --- | --- |
|  | 5 h | 10 h | 15 h | 20 h | 25 h | 30 h |
| BF29_RS01660 | 0 | 0.62 | 0.72 | 0.76 | 0.79 | 1.01 |
| BF29_RS01750 | 0 | -0.04 | 1.25 | 1.35 | 1.47 | 1.47 |
| BF29_RS02185 | 0 | -0.31 | 0.92 | 0.92 | 1.17 | 1.07 |
| BF29_RS02295 | 0 | -0.31 | 0.03 | 0.8 | 0.96 | 0.91 |
| BF29_RS02300 | 0 | -0.01 | -0.09 | 0.91 | 1.06 | 0.86 |
| BF29_RS02400 | 0 | 0.8 | 1.74 | 1.86 | 2.29 | 2.25 |
| BF29_RS02410 | 0 | 0.32 | 0.8 | 0.61 | 1.04 | 1.09 |
| BF29_RS02435 | 0 | 0.4 | 1.32 | 1.52 | 1.38 | 1.72 |
| BF29_RS02775 | 0 | -0.42 | 0.27 | 0.52 | 0.57 | 0.6 |
| BF29_RS04030 | 0 | 0.42 | 1.52 | 1.73 | 1.72 | 1.78 |
| BF29_RS04130 | 0 | 0.11 | 1.53 | 1.91 | 2.17 | 2.25 |
| BF29_RS04960 | 0 | 0.34 | 1.45 | 1.47 | 1.52 | 1.55 |
| BF29_RS04965 | 0 | -0.01 | 0.93 | 0.92 | 1.15 | 1.2 |
| BF29_RS05055 | 0 | 0.01 | 0.23 | 0.37 | 0.28 | 1.22 |
| BF29_RS05275 | 0 | 0.59 | 1.09 | 1.26 | 1.35 | 1.36 |
| BF29_RS05420 | 0 | -0.03 | 1.19 | 1.57 | 2.01 | 2.1 |
| BF29_RS05495 | 0 | 0.17 | 0.51 | 0.67 | 0.84 | 1.04 |
| BF29_RS05500 | 0 | 0.23 | 0.74 | 0.97 | 1.22 | 1.28 |
| BF29_RS05520 | 0 | -0.02 | 1.05 | 1.06 | 1.44 | 1.41 |
| BF29_RS05700 | 0 | 0.65 | 0.62 | 0.7 | 0.74 | 1.04 |
| BF29_RS05770 | 0 | 0.13 | 1.47 | 1.81 | 2.02 | 1.77 |
| BF29_RS05985 | 0 | -0.02 | 0.85 | 0.93 | 1.24 | 0.99 |
| BF29_RS06060 | 0 | -0.48 | 0.07 | 0.28 | 0.71 | 0.97 |
| BF29_RS06675 | 0 | -0.09 | 0.45 | 1.01 | 1.61 | 2.47 |
| BF29_RS06680 | 0 | 0.09 | 0.58 | 0.71 | 1.08 | 1.1 |
| BF29_RS06870 | 0 | 0.06 | 1.2 | 1.52 | 1.68 | 1.62 |
| BF29_RS06875 | 0 | -0.06 | 0.82 | 1.03 | 1.17 | 1.14 |
| BF29_RS07000 | 0 | 1.08 | 1.41 | 0.95 | 1.58 | 1.81 |
| BF29_RS07055 | 0 | 0.37 | 0.54 | 0.67 | 1.01 | 1.29 |
| BF29_RS07275 | 0 | 0.93 | 1.55 | 1.88 | 1.89 | 2.28 |
| BF29_RS07280 | 0 | -0.08 | 0.47 | 0.78 | 1.26 | 1.45 |
| BF29_RS07435 | 0 | 0.98 | 1.37 | 1.4 | 1.62 | 1.52 |
| BF29_RS07495 | 0 | 0.49 | 1.18 | 1.25 | 1.3 | 1.47 |
| BF29_RS07510 | 0 | -0.53 | 0.48 | 0.7 | 0.63 | 0.85 |
| BF29_RS07515 | 0 | -0.31 | 0.56 | 0.46 | 0.6 | 0.79 |
| BF29_RS08185 | 0 | 0.35 | 0.38 | 0.42 | 0.84 | 1.08 |
| BF29_RS08255 | 0 | 0.69 | 1.61 | 1.22 | 1.6 | 1.7 |
| BF29_RS08260 | 0 | 0.49 | 0.85 | 1.05 | 1.08 | 1.02 |
| BF29_RS08470 | 0 | 0.7 | 1.01 | 1.34 | 1.37 | 1.59 |
| BF29_RS08655 | 0 | 0.21 | 0.67 | 0.76 | 1.02 | 1.19 |
| BF29_RS08660 | 0 | 0.5 | 0.75 | 0.78 | 0.92 | 1.04 |
| BF29_RS08675 | 0 | 0.57 | 0.85 | 1.02 | 1.2 | 1.13 |
| BF29_RS08710 | 0 | 1.79 | 1.75 | 1.79 | 1.98 | 2.29 |
| BF29_RS09095 | 0 | 0.57 | 0.84 | 0.94 | 0.98 | 1.11 |
| BF29_RS09710 | 0 | 0.03 | 0.91 | 0.99 | 1.1 | 1.17 |
| BF29_RS10370 | 0 | -0.03 | 1.67 | 2 | 2.18 | 2.04 |
| BF29_RS10730 | 0 | 0.42 | 1.09 | 1.13 | 1.38 | 1.44 |
| BF29_RS10965 | 0 | -0.23 | 0.66 | 0.9 | 1.3 | 1.58 |
| BF29_RS11285 | 0 | -0.29 | 1.28 | 1.71 | 1.95 | 1.96 |
| BF29_RS11305 | 0 | 0.32 | 0.49 | 0.72 | 0.66 | 1.19 |
| BF29_RS12475, BF29_RS12145 | 0 | 0.57 | 0.74 | 0.68 | 0.98 | 1.1 |
| BF29_RS12485, BF29_RS12155 | 0 | 0.02 | 0.63 | 1.11 | 1.54 | 1.6 |
| BF29_RS12690, BF29_RS12350 | 0 | 1.22 | 1.22 | 1.31 | 1.97 | 1.64 |
| BF29_RS12945 | 0 | 0.46 | 0.82 | 0.77 | 0.89 | 1.22 |
| BF29_RS13945 | 0 | 0.18 | 1.39 | 1.78 | 1.86 | 1.97 |
| BF29_RS14265 | 0 | -0.4 | 1.54 | 2.22 | 2.5 | 2.28 |
| BF29_RS14270 | 0 | 0.83 | 2.01 | 2.79 | 3.04 | 2.64 |
| BF29_RS14620 | 0 | 0.62 | 0.81 | 0.75 | 1.13 | 1.25 |
| BF29_RS14800 | 0 | 0.01 | 0.95 | 1.08 | 1.36 | 1.42 |
| BF29_RS14805 | 0 | -0.03 | 0.68 | 0.81 | 0.91 | 1.06 |
| BF29_RS14810 | 0 | 0.28 | 1.01 | 1.21 | 1.29 | 1.34 |
| BF29_RS14830 | 0 | 0.1 | 0.62 | 0.55 | 0.85 | 1.21 |
| BF29_RS14840 | 0 | 0.52 | 0.89 | 1.07 | 1.17 | 1.2 |
| BF29_RS14875 | 0 | -0.15 | 1.46 | 1.68 | 1.85 | 2.1 |
| BF29_RS15075 | 0 | -0.22 | 0.09 | 0.27 | 0.57 | 0.93 |
| BF29_RS15500 | 0 | 0.39 | 0.92 | 0.89 | 1.16 | 1.25 |
| BF29_RS15950 | 0 | 0.31 | 0.87 | 0.78 | 1.19 | 1.6 |
| BF29_RS16055 | 0 | 0.74 | 0.96 | 1.1 | 1.17 | 1.23 |
| BF29_RS16070 | 0 | 0.52 | 0.79 | 1.11 | 1.1 | 1.38 |
| BF29_RS16590 | 0 | 0.04 | 1.7 | 2.03 | 2.23 | 2.32 |

**Table S10** TFs exhibiting an increasing trend in STEM time-series analysis.

| Locus tag | Description | log_2_(normalized value of genes) | | | | | |
| --- | --- | --- | --- | --- | --- | --- | --- |
|  |  | 5 h | 10 h | 15 h | 20 h | 25 h | 30 h |
| BF29_RS02180 | sigma-54-dependent Fis family transcriptional regulator | 0 | -0.3 | 0.34 | -0.08 | 1.84 | 1.41 |
| BF29_RS02240 | IS21-like element helper ATPase IstB | 0 | -2.02 | 1.19 | -0.1 | 0.69 | 0.94 |
| BF29_RS01940 | pyruvate, phosphate dikinase | 0 | 0.72 | 1.58 | 2.65 | 2.37 | 3.47 |
| BF29_RS02315 | LysR family transcriptional regulator | 0 | 2.03 | 3.25 | 3.48 | 3.09 | 3.21 |
| BF29_RS01460 | MarR family transcriptional regulator | 0 | -0.05 | 0.7 | 1.27 | 1.44 | 1.38 |
| BF29_RS02610 | MerR family transcriptional regulator | 0 | 0.1 | 0.44 | 0.08 | 0.7 | 2.16 |
| BF29_RS04010 | heat-inducible transcriptional repressor HrcA | 0 | 0.3 | 0.19 | 0.54 | 1.18 | 1.56 |
| BF29_RS02795 | MerR family transcriptional regulator | 0 | -0.33 | 0.57 | 1.13 | 0.24 | 2.95 |
| BF29_RS07490 | acetolactate synthase large subunit | 0 | 0.28 | 1.97 | 1.08 | 1.03 | 1.78 |
| BF29_RS06215 | helix-turn-helix domain-containing protein | 0 | 0.32 | 0.93 | 1.15 | 1.33 | 0.99 |
| BF29_RS07050 | formate C-acetyltransferase | 0 | -0.21 | -0.9 | 0.18 | 5.3 | 3.36 |
| BF29_RS05990 | metal-sensitive transcriptional regulator | 0 | -0.01 | 1.28 | 2.15 | 1.2 | 1.42 |
| BF29_RS07270 | MurR/RpiR family transcriptional regulator | 0 | 0.46 | -0.77 | -0.31 | 2.7 | 2.53 |
| BF29_RS07240 | peroxide-responsive transcriptional repressor PerR | 0 | 1.96 | 2.43 | 2.97 | 2.31 | 3.21 |
| BF29_RS07850 | MurR/RpiR family transcriptional regulator | 0 | -0.06 | 1.2 | 1.38 | 5.51 | 4.04 |
| BF29_RS13920 | helix-turn-helix domain-containing protein | 0 | 0.95 | 2.69 | 3.28 | 3.08 | 2.43 |
| BF29_RS09885 | MerR family transcriptional regulator | 0 | 0.09 | 1.83 | 1.77 | 1.06 | 1.73 |
| BF29_RS10235 | TetR/AcrR family transcriptional regulator | 0 | 0.66 | 0.92 | 1.1 | 2.39 | 2.31 |
| BF29_RS11605 | ATP-dependent protease ATP-binding subunit ClpC | 0 | 0.6 | 1.4 | 1.39 | 1.7 | 2.18 |
| BF29_RS13765 | helix-turn-helix transcriptional regulator | 0 | 0.49 | 0.92 | 0.97 | 1.29 | 1.89 |
| BF29_RS09585 | redox-sensing transcriptional repressor Rex | 0 | 0.33 | 0.34 | 0.35 | 1.91 | 1.58 |
| BF29_RS13125 | TetR family transcriptional regulator | 0 | 0.92 | 1.94 | 1.53 | 1.81 | 1.8 |
| BF29_RS13555 | response regulator | 0 | -0.06 | 1.05 | 1.16 | 1.67 | 2.73 |
| BF29_RS09325 | PadR family transcriptional regulator | 0 | 0.9 | 2.09 | 2.13 | 2.27 | 2.75 |
| BF29_RS10145 | class II fructose-bisphosphatase | 0 | 0.58 | 0.21 | 0.72 | 0.61 | 1.29 |
| BF29_RS09320 | GntR family transcriptional regulator | 0 | 0.57 | 1.24 | 1.38 | 0.87 | 1.23 |
| BF29_RS13820 | competence protein ComK | 0 | 0.13 | 2.84 | 2.8 | 2.61 | 2.56 |
| BF29_RS14205 | LacI family DNA-binding transcriptional regulator | 0 | 2.02 | 1.75 | 2.11 | 1.87 | 2.18 |
| BF29_RS08960 | transposase | 0 | 2.34 | 3.33 | 3.91 | 3.56 | 3.28 |
| BF29_RS09715 | RNA polymerase sigma factor SigB | 0 | 3.33 | 3.46 | 3.38 | 3.26 | 3.7 |
| BF29_RS11590 | CtsR family transcriptional regulator | 0 | 0.78 | 0.77 | 1.11 | 1.07 | 1.39 |
| BF29_RS14790 | sigma-54-dependent transcriptional regulator | 0 | 0.87 | 1.57 | 1.38 | 1.79 | 1.52 |
| BF29_RS15135 | metalloregulator ArsR/SmtB family transcription factor | 0 | -2.72 | -0.2 | 0.64 | 1.3 | 1.46 |
| BF29_RS15315 | GntR family transcriptional regulator | 0 | 0.08 | -0.38 | 0.12 | 1.13 | 1.03 |
| BF29_RS14625 | AraC family transcriptional regulator | 0 | 1.66 | 0.8 | 0.29 | 5.99 | 4.48 |
| BF29_RS16630 | PocR ligand-binding domain-containing protein | 0 | 0.25 | -0.47 | 0.21 | 5.38 | 3.32 |
| BF29_RS16010 | HTH-type transcriptional regulator Hpr | 0 | -0.91 | 2.05 | 2.64 | 2.64 | 2.88 |
| BF29_RS16165 | competence protein ComK | 0 | 1.1 | 2.1 | 2.16 | 2.81 | 2.43 |
| BF29_RS16095 | TetR/AcrR family transcriptional regulator | 0 | 0.49 | 0.11 | 1.63 | 2.4 | 2.41 |

**Table S11** TFs that exhibited a twofold or greater increase in gene expression levels in the 30 h samples compared with the 20 h samples.

| Locus tag | Description | Log_2_FC | P_adjust_ |
| --- | --- | --- | --- |
| BF29_RS02245 | IS21 family transposase | 6.08 | 0.00 |
| BF29_RS14625 | AraC family transcriptional regulator | 3.97 | 0.00 |
| BF29_RS07050 | formate C-acetyltransferase | 2.98 | 0.00 |
| BF29_RS16630 | PocR ligand-binding domain-containing protein | 2.89 | 0.00 |
| BF29_RS12970 | GntR family transcriptional regulator | 2.77 | 0.00 |
| BF29_RS13405 | LacI family DNA-binding transcriptional regulator | 2.75 | 0.00 |
| BF29_RS07270 | MurR/RpiR family transcriptional regulator | 2.62 | 0.00 |
| BF29_RS07850 | MurR/RpiR family transcriptional regulator | 2.44 | 0.00 |
| BF29_RS08570 | MarR family transcriptional regulator | 2.39 | 0.00 |
| BF29_RS04470 | MarR family transcriptional regulator | 2.04 | 0.00 |
| BF29_RS02610 | MerR family transcriptional regulator | 1.85 | 0.00 |
| BF29_RS07825 | response regulator transcription factor | 1.78 | 0.00 |
| BF29_RS08295 | TetR/AcrR family transcriptional regulator | 1.67 | 0.00 |
| BF29_RS00755 | ADP-forming succinate--CoA ligase subunit beta | 1.65 | 0.00 |
| BF29_RS02795 | MerR family transcriptional regulator | 1.60 | 0.00 |
| BF29_RS06530 | acetyl-CoA C-acetyltransferase | 1.51 | 0.00 |
| BF29_RS09085 | hypothetical protein | 1.45 | 0.00 |
| BF29_RS11065 | chromosomal replication initiator protein DnaA | 1.43 | 0.00 |
| BF29_RS11890 | 50S ribosomal protein L17 | 1.39 | 0.00 |
| BF29_RS12990 | Crp/Fnr family transcriptional regulator | 1.37 | 0.00 |
| BF29_RS13555 | response regulator | 1.36 | 0.00 |
| BF29_RS05070 | PadR family transcriptional regulator | 1.36 | 0.00 |
| BF29_RS04305 | Rrf2 family transcriptional regulator | 1.32 | 0.00 |
| BF29_RS07670 | MarR family transcriptional regulator | 1.31 | 0.00 |
| BF29_RS02180 | sigma-54-dependent Fis family transcriptional regulator | 1.28 | 0.01 |
| BF29_RS15850 | DNA-binding protein WhiA | 1.16 | 0.00 |
| BF29_RS03235 | response regulator transcription factor | 1.15 | 0.00 |
| BF29_RS11185 | 16S rRNA (cytidine(1402)-2'-O)-methyltransferase | 1.08 | 0.00 |
| BF29_RS04985 | endonuclease MutS2 | 1.08 | 0.00 |
| BF29_RS09340 | Rrf2 family transcriptional regulator | 1.06 | 0.00 |
| BF29_RS00645 | ATP-dependent DNA helicase RecG | 1.02 | 0.00 |
| BF29_RS09585 | redox-sensing transcriptional repressor Rex | 1.02 | 0.00 |
| BF29_RS03525 | transcriptional regulator ArgR | 1.00 | 0.00 |

**Table S12** ABC transporters showed significantly different gene expression levels between the samples collected at 25 and 20 h.

| Locus tag | Description | Log_2_FC | P_adjust_ |
| --- | --- | --- | --- |
| BF29_RS14615 | sugar ABC transporter permease | 4.72 | 0.00 |
| BF29_RS15895 | sn-glycerol-3-phosphate ABC transporter ATP-binding protein UgpC | 3.83 | 0.00 |
| BF29_RS09830 | ABC transporter permease subunit | 3.19 | 0.00 |
| BF29_RS09835 | sugar ABC transporter permease | 2.52 | 0.00 |
| BF29_RS15630 | siderophore ABC transporter substrate-binding protein | 1.79 | 0.00 |
| BF29_RS15620 | iron chelate uptake ABC transporter family permease subunit | 1.78 | 0.00 |
| BF29_RS09460 | ABC transporter ATP-binding protein | 1.72 | 0.00 |
| BF29_RS13075 | ABC transporter substrate-binding protein | 1.67 | 0.00 |
| BF29_RS01145 | BMP family ABC transporter substrate-binding protein | 1.47 | 0.00 |
| BF29_RS08565 | ABC transporter ATP-binding protein/permease | 1.36 | 0.00 |
| BF29_RS01155 | ABC transporter permease | 1.32 | 0.00 |
| BF29_RS01160 | ABC transporter permease | 1.06 | 0.00 |
| BF29_RS15615 | ABC transporter permease | 1.04 | 0.00 |
| BF29_RS06895 | ABC transporter permease | -1.24 | 0.00 |
| BF29_RS06885 | peptide ABC transporter substrate-binding protein | -1.55 | 0.00 |
| BF29_RS06890 | ABC transporter ATP-binding protein | -1.56 | 0.00 |
| BF29_RS15680 | phosphate ABC transporter permease PstA | -1.99 | 0.00 |
| BF29_RS14340 | ABC transporter ATP-binding protein | -2.03 | 0.01 |
| BF29_RS14240 | betaine/proline/choline family ABC transporter ATP-binding protein | -2.25 | 0.00 |
| BF29_RS15675 | phosphate ABC transporter permease subunit PstC | -2.26 | 0.00 |
| BF29_RS14225 | ABC transporter permease | -2.27 | 0.00 |
| BF29_RS14235 | ABC transporter permease | -2.34 | 0.00 |
| BF29_RS10690 | ABC transporter substrate-binding protein | -2.36 | 0.00 |
| BF29_RS15685 | phosphate ABC transporter ATP-binding protein PstB | -2.46 | 0.00 |
| BF29_RS14230 | osmoprotectant ABC transporter substrate-binding protein | -2.71 | 0.00 |
| BF29_RS15690 | phosphate ABC transporter ATP-binding protein PstB | -2.85 | 0.00 |
| BF29_RS10680 | sugar ABC transporter permease | -2.95 | 0.00 |
| BF29_RS10685 | carbohydrate ABC transporter permease | -2.97 | 0.00 |
| BF29_RS15670 | phosphate ABC transporter substrate-binding protein PstS family protein | -3.00 | 0.00 |
| BF29_RS10675 | ABC transporter ATP-binding protein | -3.10 | 0.00 |

**Table S13** ABC transporters showed significantly different gene expression levels between the samples collected at 30 and 25 h.

| Locus tag | Description | Log_2_FC | P_adjust_ |
| --- | --- | --- | --- |
| BF29_RS14225 | ABC transporter permease | 1.86 | 0.00 |
| BF29_RS14235 | ABC transporter permease | 1.73 | 0.00 |
| BF29_RS14230 | osmoprotectant ABC transporter substrate-binding protein | 1.73 | 0.00 |
| BF29_RS14240 | betaine/proline/choline family ABC transporter ATP-binding protein | 1.47 | 0.00 |
| BF29_RS13065 | iron ABC transporter permease | 1.33 | 0.00 |
| BF29_RS06885 | peptide ABC transporter substrate-binding protein | 1.30 | 0.00 |
| BF29_RS06890 | ABC transporter ATP-binding protein | 1.08 | 0.00 |
| BF29_RS10585 | ABC transporter permease | 1.07 | 0.00 |
| BF29_RS01145 | BMP family ABC transporter substrate-binding protein | -1.07 | 0.00 |
| BF29_RS09835 | sugar ABC transporter permease | -1.14 | 0.00 |
| BF29_RS15895 | sn-glycerol-3-phosphate ABC transporter ATP-binding protein UgpC | -1.70 | 0.00 |
| BF29_RS10690 | ABC transporter substrate-binding protein | -3.10 | 0.00 |
| BF29_RS10685 | carbohydrate ABC transporter permease | -3.60 | 0.00 |
| BF29_RS15680 | phosphate ABC transporter permease PstA | -3.83 | 0.00 |
| BF29_RS10675 | ABC transporter ATP-binding protein | -4.26 | 0.00 |
| BF29_RS15690 | phosphate ABC transporter ATP-binding protein PstB | -4.55 | 0.00 |
| BF29_RS10680 | sugar ABC transporter permease | -4.72 | 0.00 |
| BF29_RS15685 | phosphate ABC transporter ATP-binding protein PstB | -4.82 | 0.00 |
| BF29_RS15675 | phosphate ABC transporter permease subunit PstC | -4.88 | 0.00 |
| BF29_RS15670 | phosphate ABC transporter substrate-binding protein PstS family protein | -4.88 | 0.00 |

**Table S14** ABC transporters showed significantly different gene expression levels between the samples collected at 30 and 20 h.

| Locus tag | Description | Log_2_FC | P_adjust_ |
| --- | --- | --- | --- |
| BF29_RS14615 | sugar ABC transporter permease | 3.70 | 0.02 |
| BF29_RS15630 | siderophore ABC transporter substrate-binding protein | 2.66 | 0.00 |
| BF29_RS15620 | iron chelate uptake ABC transporter family permease subunit | 2.53 | 0.00 |
| BF29_RS09830 | ABC transporter permease subunit | 2.34 | 0.00 |
| BF29_RS15895 | sn-glycerol-3-phosphate ABC transporter ATP-binding protein UgpC | 2.13 | 0.00 |
| BF29_RS08565 | ABC transporter ATP-binding protein/permease | 1.90 | 0.00 |
| BF29_RS13065 | iron ABC transporter permease | 1.66 | 0.00 |
| BF29_RS01155 | ABC transporter permease | 1.57 | 0.00 |
| BF29_RS13485 | ABC transporter permease | 1.50 | 0.00 |
| BF29_RS13075 | ABC transporter substrate-binding protein | 1.48 | 0.00 |
| BF29_RS09835 | sugar ABC transporter permease | 1.38 | 0.00 |
| BF29_RS08560 | ABC transporter ATP-binding protein/permease | 1.18 | 0.00 |
| BF29_RS15615 | ABC transporter permease | 1.14 | 0.00 |
| BF29_RS10585 | ABC transporter permease | 1.03 | 0.00 |
| BF29_RS01160 | ABC transporter permease | 1.03 | 0.00 |
| BF29_RS08300 | ABC transporter permease | 1.01 | 0.00 |
| BF29_RS10690 | ABC transporter substrate-binding protein | -5.46 | 0.00 |
| BF29_RS15680 | phosphate ABC transporter permease PstA | -5.82 | 0.00 |
| BF29_RS10685 | carbohydrate ABC transporter permease | -6.56 | 0.00 |
| BF29_RS15675 | phosphate ABC transporter permease subunit PstC | -7.14 | 0.00 |
| BF29_RS15685 | phosphate ABC transporter ATP-binding protein PstB | -7.28 | 0.00 |
| BF29_RS10675 | ABC transporter ATP-binding protein | -7.36 | 0.00 |
| BF29_RS15690 | phosphate ABC transporter ATP-binding protein PstB | -7.40 | 0.00 |
| BF29_RS10680 | sugar ABC transporter permease | -7.67 | 0.00 |
| BF29_RS15670 | phosphate ABC transporter substrate-binding protein PstS family protein | -7.88 | 0.00 |


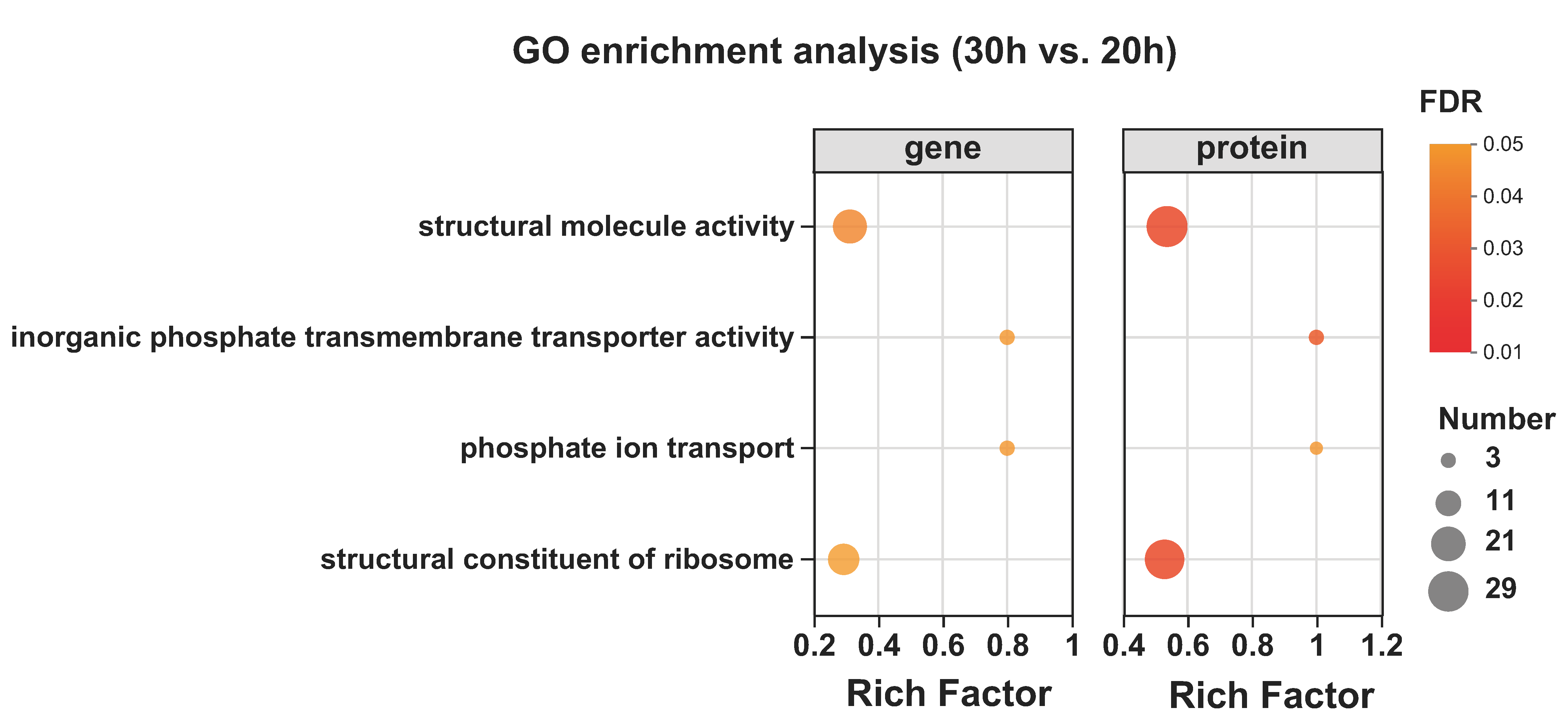


**Fig. S1** Significantly enriched GO terms between the 30 and 20 h samples in both transcriptome and proteomics analyses.
